# QUANTITY: An Isobaric Tag for Quantitative Glycomics

**DOI:** 10.1038/srep17585

**Published:** 2015-11-30

**Authors:** Shuang Yang, Meiyao Wang, Lijun Chen, Bojiao Yin, Guoqiang Song, Illarion V. Turko, Karen W. Phinney, Michael J. Betenbaugh, Hui Zhang, Shuwei Li

**Affiliations:** 1 Department of Pathology, Johns Hopkins University, Baltimore, MD, USA; 2 Institute for Bioscience and Biotechnology Research, University of Maryland College Park, Rockville, MD, USA; 3 Department of Chemical and Biomolecular Engineering, Johns Hopkins University, Baltimore, MD, USA; 4 School of Pharmaceutical Engineering and Life Science, Changzhou University, Changzhou, China; 5 Biomolecular Measurement Division, National Institute of Standard and Technology, Gaithersburg, MD, USA; 6 Analytical Biotechnology, MedImmune LLC, Gaithersburg, MD, USA

## Abstract

Glycan is an important class of macromolecules that play numerous biological functions. Quantitative glycomics - analysis of glycans at global level - however, is far behind genomics and proteomics owing to technical challenges associated with their chemical properties and structural complexity. As a result, technologies that can facilitate global glycan analysis are highly sought after. Here, we present QUANTITY (Quaternary Amine Containing Isobaric Tag for Glycan), a quantitative approach that can not only enhance detection of glycans by mass spectrometry, but also allow high-throughput glycomic analysis from multiple biological samples. This robust tool enabled us to accomplish glycomic survey of bioengineered Chinese Hamster Ovary (CHO) cells with knock-in/out enzymes involved in protein glycosylation. Our results demonstrated QUANTITY is an invaluable technique for glycan analysis and bioengineering.

Glycans (a.k.a. carbohydrate or polysaccharide) are an important class of biomolecules that play critical roles in biological processes such as protein trafficking, cell-to-cell communication and immune responses, and their abnormality could be associated with numerous diseases including cancer, dementia, and autoimmune disorders^1^,^2^. The potency and stability of therapeutic biological drugs like monoclonal antibodies are also affected by glycans they carry^3^. As a result, understanding glycan functions is of great significance in academic research, pharmaceutical industry and healthcare^4^. However, the progress on glycan research is far behind other biomolecules like nucleic acids and proteins because of technical challenges associated with their unique chemical properties and structural complexity^5^.

To assist structural analysis, glycans are usually derivatized (e.g. fluorescence tags like 2-aminobenzamide (2-AB) or 2-aminobenzoic acid (2-AA), permethylation, etc.) to improve their characterization on various analytical platforms, including capillary electrophoresis (CE), liquid chromatography (LC), mass spectrometry (MS), and so on^6^. MS has become one of the most popular tools for glycan analysis mainly because of its ability to determine glycan compositions even from overlapping peaks and its compatibility with other methods (e.g. RapFluor-MS^7^). Meanwhile, stable isotope labels have also gained increasingly popularity as they allow accurate quantification of glycans using MS.

There are two major types of stable isotope labeling approaches, mass shift and isobaric tags, both of which have been extensively used for the quantification of proteins^8^,^9^,^10^ and small molecules such as metabolites^11^,^12^. A primary difference between mass shift and isobaric tags is that quantitation of a mass shift tag is achieved in MS^1^ while isobaric quantification relies on reporter ions generated in MS^2^ or MS^3^. Despite multiple advantages offered by isobaric tags, such as allowing quantification of up to ten samples in a single assay^13^ and increasing detection limit by accumulating signals from multiple samples together, there are very few successes to develop isobaric tags for glycan quantification. In fact, to our best knowledge, only two isobaric tags, aminoxyTMT^14^ and iART^15^ from our own work, have been applied for glycan quantification and shown limited success. One of reasons is that both isobaric tags were based on a tertiary amine structure that was originally designed for peptide quantification and fragments less favorably than glycosidic bonds in MS^2^. Therefore, neither aminoxyTMT nor iART can generate reporter ions strong enough for accurate quantification of labeled glycans, especially for high molecular weight glycans.

Here, we report a novel type of isobaric tags capable of fragmenting as easily as glycosidic bonds, which is based on our serendipitous discovery that a quaternary amine can easily lose one of its four substituents on nitrogen upon MS^2^ fragmentation. The tags, termed Quaternary Amine Containing Isobaric Tag for Glycan (QUANTITY), can completely label glycans and generate strong reporter ions. Up to four samples can be labeled and analyzed concurrently for the relative quantification of glycans. To demonstrate the application of QUANTITY, we examined the protein N-glycosylation of several genetically engineered Chinese Hamster Ovary (CHO) cell lines, in which one of glycosyltransferases was either knocked in or knocked out. To our best knowledge, this is the first quantitative glycomic analysis of engineered CHO cells. Since CHO cells are used for the production of monoclonal antibodies in the pharmaceutical industry, our results would pave a way for the better understanding of glycosylation on therapeutic proteins and lead to more potent biological drugs with desirable pharmaceutical properties.

## Results

### Design of QUANTITY

**Figure 1:**
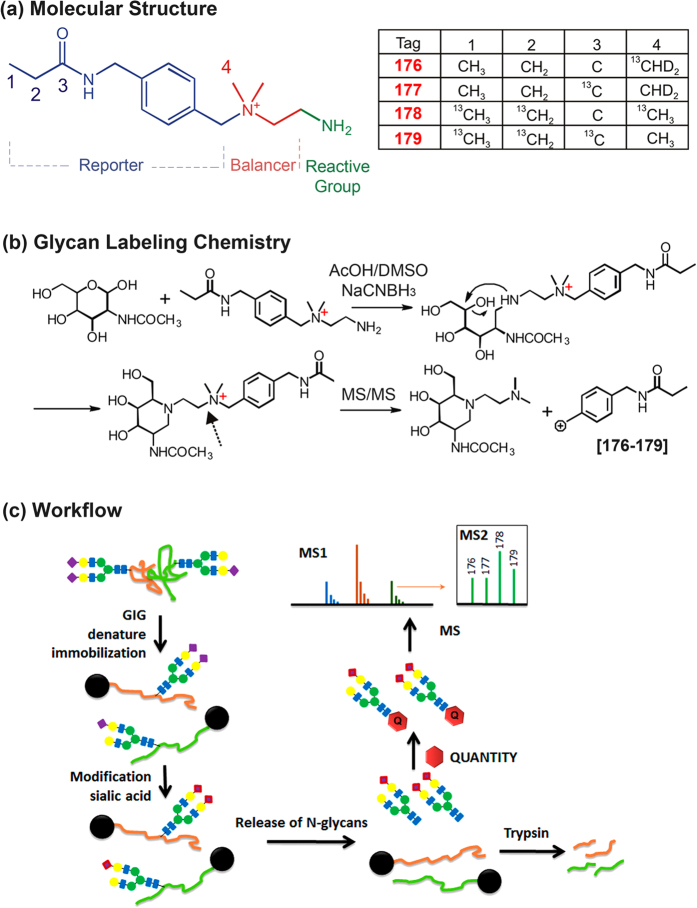
QUANTITY isobaric tandem mass tags for glycan labeling and quantitation. (**a**) Molecular structure and isotope positions of QUANTITY. (**b**) N-acetylglucosamine (GlcNAc, the first residue on the reducing end of N-glycans) labeled with QUANTITY and its fragmentation in MS^2^. A water molecule is lost during labeling, probably through a six-membered ring formation (neighboring group participation reaction). The arrow indicates fragmentation site in MS^2^. (**c**) Workflow of N-glycan extraction from solid-phase for labeling of N-glycans by isobaric tandem mass tags and analysis of peptide using LC-MS/MS. Proteins are immobilized on beads via reductive amination. Sialic acids are stabilized via carbodiimide coupling. The released N-glycans by PNGase F are labeled with QUANTITY. The global proteins are further analyzed by direct digestion from beads.

As other isobaric tags for peptides and small molecules, the 4-plex QUANTITY reagents are a set of four molecules with identical chemical structures and molecule weight, yet they contain different stable isotope nuclei like ^13^C and ^2^H in various positions (Synthesis of QUANTITY is described in Supporting Materials Figures S1&2). Their structures consist of a reporter with molecular mass ranging from 176 to 179 Daltons in the series, a balancer that compensates the mass difference of the reporters, and a reactive primary amine to conjugate with glycans via reductive amination (Fig. 1). This labeling chemistry is the same as that used by 2-AA/2-AB^16^, so well-established protocols for 2-AA/2-AB labeling can be applied without much modification. A noticeable difference between our tags and 2-AA/2-AB, however, is that a water molecule is lost spontaneously and stoichiometrically from QUANTITY-labeled glycans, while 2-AA/2-AB labeled glycans only show the partial loss of a water molecule^17^. This phenomenon might be proceeded through an energetically favored six-membered ring formation in a neighboring group participation mechanism (a.k.a. neighboring participation reaction)^18^. Upon MS^2^ fragmentation, QUANTITY-labeled glycans yield strong reporter ions for accurate quantification without the need of extra positive ions such as Na^+^ or metal ions, thereby eliminating ion suppression effect and preventing the formation of multiple H^+^/Na^+^ adducts. Furthermore, outfitting glycans with a permanently positive-charged quaternary amine can enhance their ionization in MS. Therefore, the detection sensitivity of glycans is considerably enhanced, which is advantageous when analyzing low abundance glycans or limited samples.

To apply QUANTITY for glycan analysis, we have developed a solid-phase based protocol (Fig. 1)^15^. Glycoproteins are first immobilized on beads and treated with excess p-toluidine (pT) in the presence of a carbodiimide coupling reagent. This step can completely conjugate sialic acids on glycans with pT to stabilize these labile residues during MS analysis. N-glycans are then released from the immobilized glycoproteins with PNGase F, resulting in an exposed aldehyde group at their reducing end for QUANTITY labeling. The labeled glycans are then analyzed with reverse phase liquid chromatography (RPLC) coupled with tandem MS. A bonus of this protocol is that a hydrophobic pT moiety is coupled to each sialic acid, so N-glycans carrying different number of sialic acids can be easily resolved on a C18 column.

The monoisotopic mass of a labeled glycan, M = F_*a*_N_*b*_H_*c*_S_*d*_G_*e*_, is calculated based on *Equation 1*.


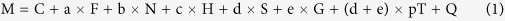


Here: C stands for N-glycan core structure, 910.3278 Da; F is fucose, 146.0579 Da; N is HexNAc, 203.0794 Da; H is Hexose, 162.0528 Da; S is Neu5Ac, 291.0954 Da; G is Neu5Gc, 307.0903 Da; pT is p-Toluidine, 89.0629 Da (after the loss of one water), which is coupled to each sialic acid in our protocol; Q is QUANTITY, 233.2147 Da (after the loss of one oxygen due to reductive amination and the loss of one water due to neighboring participation reaction); a, b, c, d, and e is the number of each respective unit. It is important to note that the core structure (2 HexNAc and 3 Hexose) is excluded from the formula (F_*a*_N_*b*_H_*c*_S_*d*_G_*e*_) of N-glycans throughout this report. In other words, b and c represent the HexNAc and Hexose units other than those in the core structure, respectively.

In ESI, a labeled glycan is usually detected as an ion carrying multiple charges (Z) with observed m/z as B. We can calculate its monoisotopic mass (M′) based on *Equation 2*.


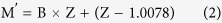


To identify a glycan from an MS experiment, we can first calculate the monoisotopic mass (M) of all QUANTITY labeled N-glycans in a glycan library, such as Consortium for Functional Glycomics (CFG) database. Then, we can calculate the monoisotopic mass (M′) of an ion in an MS^1^ spectrum and match with the monoisotopic mass (M) calculated from the glycan database to determine its composition.

### Performance of QUANTITY

**Figure 2:**
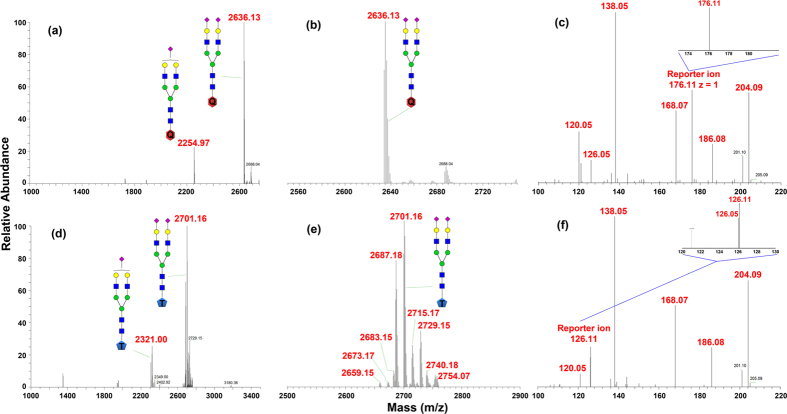
Comparison of N-glycan labeling by QUANTITY and aminoxyTMT-126 (TMT). Standard sialylglycopeptide (SGP) was used for labeling via reduction amination (1M NaCHBH_3_, DMSO:HOAc = 7:3, 65 °C/4h). SGP has two sialic acids including N_2_H_2_S and N_2_H_2_S_2_. **(a)** Full MS spectrum of SGP-QUANTITY. **(b)** MS^1^ spectrum of N_2_H_2_S_2_-QUANTITY. **(c)** MS^2^ reporter ions of N_2_H_2_S_2_-QUANTITY. **(d)** Full MS spectrum of SGP-TMT. **(e)** MS^1^ spectrum of N_2_H_2_S_2_-TMT, several by-products observed after aminoxyTMT labeling. (**f**) MS^2^ reporter ions of N_2_H_2_S_2_-TMT.

We first demonstrated the completion of QUANTITY labeling. Two N-glycans (N_2_H_2_S_2_ > 90%, and N_2_H_2_S < 10%) extracted from 1 mg standard sialylglycopeptide (SGP) were labeled with QUANTITY-176 (30 μL at 40 mM) in 70% dimethyl sulfoxide (DMSO) and 30% acetic acid (HOAc) containing 1 M sodium cyanoborohydride (NaCNBH_3_). As a comparison, the SGP glycans were also labeled by a commercially available isobaric tag for glycans based on tertiary amine (aminoxyTMT-126) similarly. Figure 2 shows the MS^1^ (a & b) and MS^2^ (c) of QUANTITY-labeled SGP glycans, as well as the MS^1^ (d & e) and MS^2^ (f) of aminoxyTMT labeled counterparts acquired on an ESI instrument (their MALDI MS^1^ spectra, together with the MALDI MS^1^ of unlabeled SGP glycans, are also available in Figure S4). The QUANTITY-labeled glycans only show two dominant peaks as labeled N_2_H_2_S and N_2_H_2_S_2_, indicating the labeling reaction was completed. Although aminoxyTMT-labeled glycans also show two major peaks, we still observed unlabeled N_2_H_2_S and N_2_H_2_S_2_, suggesting the labeling reaction with aminoxyTMT was more difficult to complete. In addition, the aminoxyTMT labeled glycans display multiple satellite peaks with 14 Dalton mass difference (Fig. 2e), making MS^1^ more complex and averaging out peak intensity. More significantly, when the labeled glycans were fragmented, the reporter ion (m/z 176.11) generated from the quaternary amine of QUANTITY yields decent signal in comparison to common glycan fragment ions (m/z 138.05 or 204.09). In contrast, the reporter ion (m/z 126.11) generated from the tertiary amine of aminoxyTMT not only shows much less intensity, but is susceptible to the interference from a glycan fragment (m/z 126.05) (Fig. 2c,f). This experiment clearly demonstrated the completion of QUANTITY labeling and its advantages over the existing isobaric tags for glycans.

**Figure 3:**
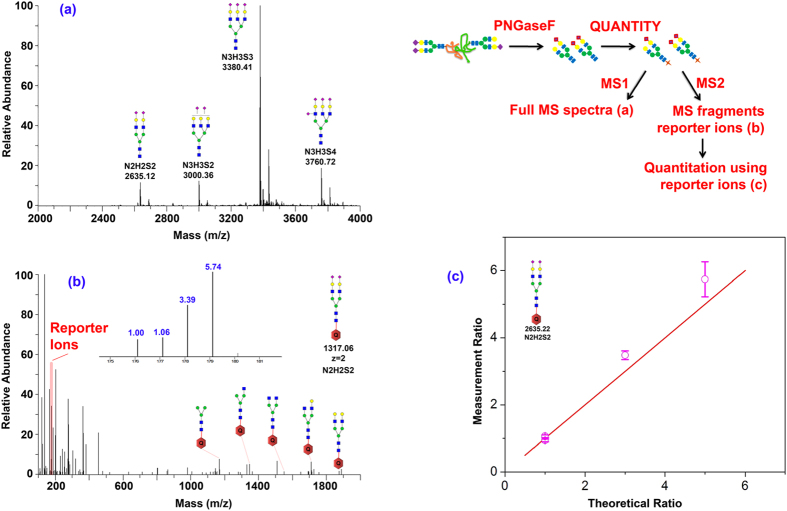
Efficiency and linear range of glycan labeling with QUANTITY. N-Glycans extracted from bovine fetuin are prepared in a ratio of 1:1:3:5 prior to labeling with QUANTITY via reduction amination. The labeled fetuin glycans (m/z at 176, 177, 178, and 179), sialylated glycans, including N_2_H_2_S_2_, N_3_H_3_S_2_, N_3_H_3_S_3_, and N_3_H_3_S_4_, are pooled for electrospray (ESI) – tandem mass spectrometry (MS^2^) (note: core structure N_2_H_3_ is not included in the composition). (**a**) MS spectrum of fetuin N-glycans. (**b)** MS^2^ spectra of N_2_H_2_S_2_ consist of glycan fragmentation and QUANTITY reporter ions. (**c**) Linear range of glycan-QUANTITY labeling at a ratio of 1:1:3:5. Error bar were obtained from three independent experiments.

Next, we tested the quantification accuracy of QUANTITY by labeling fetuin N-glycans with 4-plex QUANTITY at a ratio of 1:1:3:5. Fetuin proteins from fetal bovine (200 μg, 200 μg, 600 μg, and 1000 μg; triplicates) were processed by following our standard protocol. Figure 3a shows the MS^1^ of QUANTITY labeled fetuin N-glycans, including N_2_H_2_S_2_, N_3_H_3_S_2_, N_3_H_3_S_3_, and N_3_H_3_S_4_. Figure 3b is a representative full MS^2^ spectrum (N_2_H_2_S_2_), which includes a series of glycan fragments for easy structural elucidation of the precursor ion and strong reporter ions ranging from 176 to 179. The inset is the expanded low mass range of the MS^2^ showing the signal of each reporter ion, which indicates the relative abundance of glycans from four original samples. The experimental result of this glycan (N_2_H_2_S_2_) is very close to 1:1:3:5 (Fig. 3c). The linear correlation between the measured and theoretical ratios and the small standard deviation from three independent replicates indicate the great reproducibility of QUANTITY quantification (the quantification of other glycans is provided in the supporting information Table S1).

We then tested whether QUANTITY can be applied for complex biological samples. N-Glycans from 20 μL human serum were extracted and labeled with QUANTITY completely. A quick survey with MALDI-MS showed the profiling of serum N-glycans after QUANTITY labeling was very similar to that of unlabeled native N-glycans (Figure S5), whereas a more detailed analysis with RPLC-MS allowed us to identify over 90 N-glycans from serum (Table S3). We provide an example on how to assign a glycan structure based on its precursor mass and MS^2^ fragments (Table S2 and Figure S6). Clearly, QUANTITY is a robust and sensitive approach for glycan analysis from complex biological specimens.

### Glycomic Investigation on Bioengineered CHO Cells

In 2014, seven of the ten best-selling drugs are recombinant proteins that treat various conditions including cancer, diabetes and arthritis. These therapeutic proteins, especially monoclonal antibodies, are usually modified with N-glycans, which have profound impact on their efficacy and stability. CHO cells are widely used for the production of these biological drugs since they can produce glycoproteins compatible with humans and glycosylation can also be manipulated through genetic engineering^19^,^20^. It is well known that the modification of glycan biosynthetic pathways can alter the size of the glycans and the sites available for sialic acid attachment, which are associated with circulatory half-life of many therapeutic glycoproteins like erythropoietin (EPO)^21^,^22^. Therefore, it is of great significance for biopharmaceutical industry to investigate protein glycosylation on normal and genetically engineered CHO cells. To demonstrate the feasibility of QUANTITY for this important application, we quantitatively analyzed N-glycans from three different CHO cells, including wild-type CHO-K1 (WT), CHO-K1 with a knock-in ST6Gal1 gene (ST6Gal1(+)), and CHO-K1 with a knock-out ST3Gal4 gene (ST3Gal4(-)). ST6Gal1 and ST3Gal4 represent ST6 beta-galactosamide alpha-2,6-sialyltranferase 1 and ST3 beta-galactoside alpha-2,3-sialyltransferase 4 respectively, both of which belong to the glycosyltransferase 29 family that is involved in protein glycosylation.

**Figure 4:**
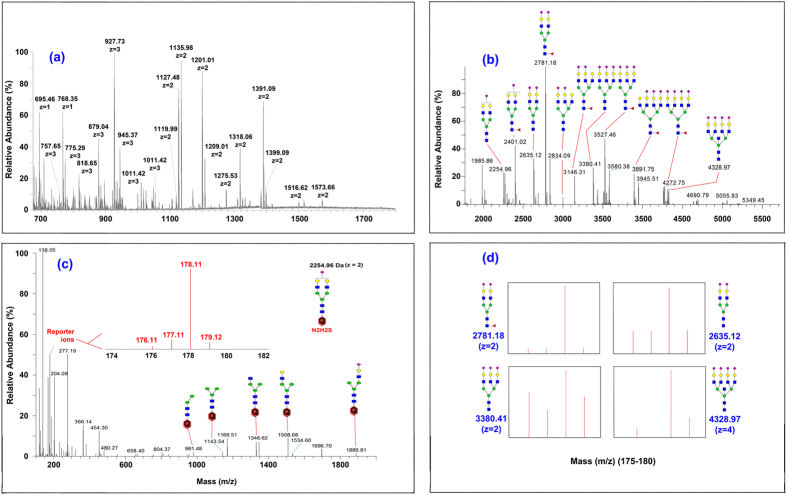
MS profiling and MS/MS quantitation of QUANTITY-labeled glycans from ST6Gal1(+) and ST3Gal4(−) CHO cells. N-Glycans are extracted using method described in Fig. 1. (**a**) MS^1^ spectrum of CHO glycans with multiple charges. **(b)** MS^1^ spectrum of CHO glycans after converting to single charge (Xcalibur). **(c)** MS^2^ spectrum of one sialylated glycan, N_2_H_2_S. **(d)** Quantitation using reporter ions from four QUANTITY-labeled glycans.

We cultured three cell lines in F12-K medium with fetal bovine serum, except for ST6Gal1(+) in which blasticidin was also included. Glycans extracted from these cells were analyzed by using our standard solid phase based protocol (Figure S8)^23^. Most QUANTITY-labeled glycans exhibited multiple charges (+2 and +3) and gave satisfactory MS^2^ fragments for structural determination (Fig. 4a,c). After all precursor ions were extracted as singly-charged peaks by Thermo Xcalibur-Xtract (Fig. 4b), each glycan only consisted of a single species and no metal adducts were observed. This feature, unique to QUANTITY-labeled glycans, was significant in that glycans could otherwise form multiple metal adducts that average out their intensity to more species and reduce their detection sensitivity. Figure 4c is the representative full MS^2^ spectrum of N_2_H_2_S showing strong signal on reporter ions for its quantification and distinct large fragments for its structural identification. The reporter ions of several other glycans, including FN_2_H_2_S_2_ (2782.2), N_2_H_2_S_2_ (2635.1), N_3_H_3_S_3_ (3380.4), and N_5_H_4_S_4_ (4329.0), are also shown in Fig. 4d.

**Figure 5:**
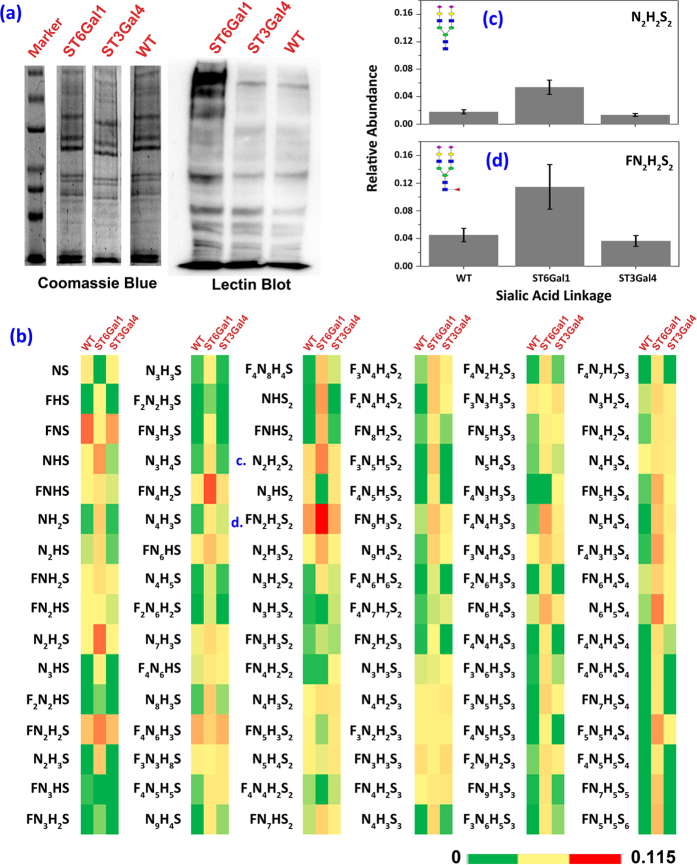
Regulation of sialic acid in CHO by ST6Gal1(+) and ST3Gal4(−). Same amount of proteins are used for glycan extraction and labeling. (**a**) Coomassie blue of CHO cell proteins on WT, ST6Gal1(+), and ST3Gal4(−); Lectin blot on WT and ST6Gal1 indicates increased sialic acid expression in ST6Gal1 knock-in CHO cells. (**b**) Heatmap of sialylated N-glycans from CHO cell glycoproteins on WT, ST6Gal1(+), and ST3Gal4(−). Quantitation is obtained from MS^2^ of QUANTITY-labeled N-glycans. Increase of sialic acid expression is observed in ST6Gal1(+) while down-regulated expression is observed in ST3Gal4(−), such as **(c)** N_2_H_2_S_2_, (**d**) FN_2_H_2_S_2_.

A complete list of N-glycans from CHO (WT, ST6Gal1(+), and ST3Gal4(−)) is provided (Fig. 5, Table S4 and Figure S8 & S9). Lectin blot confirmed the up-regulation of sialic acids in ST6Gal1 (+) and the partial down-regulation of sialic acids in ST3Gal4 (−) (Fig. 5a). A total of 159 N-glycans were quantitatively analyzed, in which 114 (71.2%) of the N-glycans were up-regulated in ST6Gal1(+). These over-expressed N-glycans had a greater number of terminal sialic acids, as depicted in the heatmap (Fig. 5b). The most abundant sialylated glycans, including N_2_H_2_S, N_2_H_2_S_2_, FN_2_H_2_S_2_, are over-expressed in ST6Gal1(+) while down-regulated in ST3Gal4(−) as expected (Fig. 5c–e). In addition, 44 glycans were only detected in ST6Gal1(+) compared to WT, such as FN_5_H_5_S_6_. In contrast, only 22 (13.8%) glycans were down-regulated in ST3Gal4(−). Three of them (FN_2_H_2_S_3_, FH_3_, and FN_3_HS) were completely absent in ST3Gal4(−), while the other 19 glycans (e.g. N_3_H_3_S, N_2_H_2_S_2_, FN_2_H_2_S_2_, and FN_6_H_2_) were only down-regulated, probably because other enzymes partially compensate the lost ST3Gal4 activity. These results indicate that protein glycosylation in CHO cells can be significantly modified by regulating the expression of a large family of genes that are involved in glycan biosynthesis. This can not only provide a powerful means to quantitatively investigate the *in vivo* functions of these enzymes, but also lead to engineered CHO cells for the production of therapeutic proteins with desirable pharmaceutical properties.

## Discussion

QUANTITY is the first quaternary amine based isobaric tag for glycan quantification and uses the same chemistry as popular 2-AA/2-AB. Because quaternary amine is permanently positive-charged, QUANTITY-labeled glycans ionize easily in MS, which improves their detection sensitivity significantly.

Even though isobaric tags have been widely used for the quantification of peptides and small molecule metabolites, previous attempts for glycan quantification have achieved limited success^14^,^15^,^24^. For example, aminoxyTMT-labeled glycans need to form metal ion adducts to show reporter ions strong enough for quantification upon fragmentation, which can cause ion suppression in MS and reduce detection sensitivity (personal communication). This is not surprising because aminoxyTMT and other isobaric tags based on tertiary amine were originally designed for peptide quantification. Since glycosidic bonds in glycans fragment much easier than peptide bonds, aminoxyTMT-labeled glycans preferably break apart between glycan units and are therefore inefficient to generate reporter ions. Our discovery that quaternary amine fragments as easily as glycosidic bonds in MS^2^ has made it possible to develop QUANTITY, which enabled us to achieve global profiling of N-glycans from up to four samples simultaneously for the first time.

Using QUANTITY, we compared the glycomics of three CHO cell lines, including WT, ST6Gal1(+), and ST3Gal4(−). CHO cells are the most widely used cell line for the production of recombinant therapeutic proteins. As glycosylation can alter a number of *in vivo* properties of glycoprotein, engineering mammalian cells with various enzymatic activities of the glycosylation pathway to alter the pattern of glycoforms is highly desired for a number of biotechnology applications, including monoclonal antibodies and other therapeutic modalities. We found the over-expression of ST6Gal1 indeed boosted protein N-glycosylation and led to increased level of sialylation, while the under-expression of ST3Gal4 showed opposite effect to less degree.

Our results indicate QUANTITY is a robust tool to investigate the biological functions of protein glycosylation and understand their roles in drug discovery and development. For example, the terminal sialic acids on N-glycans are well known for their contribution to biological characteristics of many glycoproteins, such as stability, solubility, degradation, and antigenicity. They can prevent glycoproteins from being recognized and removed by the asialoglycoprotein receptor of hepatocyte cells to improve their circulatory lifespan^22^,^25^. Thus, maximizing sialic acid content in therapeutic glycoproteins is highly desirable by pharmaceutical industry to ensure their quality and consistency. Our study therefore demonstrates the power of glycomic analysis and provides a guideline for the bioengineering of CHO cells for better protein drug production.

Because glycans, along with their sialylation level, have such important biological functions, we believe QUANTITY, integrated with solid-phase based glycoprotein immobilization for glycopeptide and glycan (GIG)^26^,^27^, would be a robust tool for both academic research and industrial applications.

## Methods

### Glycan enrichment using GIG

#### Materials and Reagents

Sialylglycopeptide (SGP) was purchased from Fushimi Pharmaceutical Co., Ltd. (Marugame, Kagawa, Japan). Spin columns (snap cap), AminoLink resin, aminoxyTMTzero, and Zebra desalting columns were purchased from Pierce (Thermo Fisher Scientific Inc.; Rockford, IL); Carbograph was purchased from Grace (Deerfield, IL). Peptide-N-glycosidase F (PNGase F), denaturing buffer, and reaction buffer G7 were from New England BioLabs (Ipswich, MA). F12K (500 mL), FBS (50 mL), NEAA (5 mL), L-glutamine (5 mL), and Blasticidin are purchased from Life Technologies (Frederick, MD). Fetuin from fetal bovine serum, p-Toluidine (pT), 2,5-dihydroxybenzoic acid (DHB), and N,N-dimethylaniline (DMA) were purchased from Sigma-Aldrich (St. Louis, MO); μ-Focus MALDI plate and its holder were form Hudson Surface Technology (Forte Lee, NJ); Axima Resonance MALDI QIT-TOF mass spectrometry was from Shimadzu Biotech (Columbia, MD). Human sera were collected from healthy men with the approval of the Institutional Review Board of Johns Hopkins University and pooled for use. Experiments were carried out in accordance with the IRB approved guidelines; all experimental protocols were approved by the IRB of Johns Hopkins University; informed consent was obtained from all subjects. All other chemicals were purchased from Sigma unless specified otherwise.

#### Wild-Type, ST6Gal1 knockin, and ST3Gal4 knockdown of Chinese Hamster Ovary (CHO) cell lines

CHO-K1 cells were recently purchased from Life Technologies. All cell lines were grown in F12-K medium (Gibco) supplemented with 10% fetal bovine serum (FBS) (Life Technologies) in a humidified 37 °C incubator with 5% CO2. Cells were seeded into a 6-well plate at appropriate densities and transfected 24 h later using Lipofactamine 2000 (Life Technologies), according to the manufacturer’s instruction.

Stable single clones were screened using select drugs, followed by lectin blot to evaluate the effect of glycosyltransferase expression. As for ST6 plasmid: ST6Gal1 (PubMed Gene ID: 6480) cDNA was purchased from OriGene and subcloned into pEF6/V5-his TOPO TA. For ST3Gal4: there are 6 members in ST3 family, we used Crisper to target ST3Gal4 gene in this family (unpublished data).

Before starting the protocol, cell media, PBS, and trypsin were placed in a 37 °C humidified oven. The sterile dishes (10 cm in diameter) were used for cell culture. Cells were quickly thawing in 37 °C water bath. Cells were transferred to 10 cm sterile dish and washed with F12-K (10% FBS, 1% NEAA, and 1% L-glutamine; note add blasticidin (250 μg in 500 mL F12-K) only for ST6Gal1). Cells were washed using 10 mL 1x PBS (6×) before being collected for cell lysate.

#### Protein immobilization and sialic acid derivatization

Cells were first sonicated for 30 s in RIPA buffer at an interval of 30 s on ice for a total of 3 minutes. The RIPA buffer (Life Technologies) of proteins was exchanged with pH 10 buffer using Zebra desalting column (Thermo). Protein concentration was measured by NanoDrop Lite Spectrophotometer (Thermo). Proteins or peptides were conjugated to beads using reductive amination. For SGP (1 mg), 200 μL of AminoLink resin (500 μL of 50% slurry) was incubated with sample in 500 μL buffer (pH 10.0) (100 mM sodium citrate and 50 mM sodium carbonate) at room temperature for 4 h with mixing. Then 50 μL of 500 mM NaCNBH3 in DI was added to incubate for another 4 h. After rinsing the resin with 500 μL of 50 mM phosphate buffer (1 × pH 7.4) twice, sample on beads were further reduced by adding 50 mM NaCNBH3 in 50 mM PBS at room temperature for 4 h with mixing. After incubation, the beads were washed with 1 M Tris-HCl (500 μL, pH 7.6) twice before addition of 500 μL of 1 M Tris-HCl (pH 7.6) in the presence of 50 mM NaCNBH3 to block the unreacted aldehyde sites on the bead surface for 0.5 h. For protein conjugation, 200 μg of proteins from each CHO cell line, 2 mg fetuin or 20 μL of serum proteins were first denatured in 100 μL solution consisting of 10× denaturing buffer (New England Biolabs) and 90 μL buffer (pH 10.0) for 10 minutes at 100°C before following same protocol as that of SGP. The immobilized samples were washed three times with 500 μL of 1 M NaCl and three times with H2O.

To protect the sialic acid groups, glycans on the solid support were incubated with 465 μL of p-toluidine (Sigma) solution (pH 4–6), which consists of 400 μL p-toluidine, 25 μL 36–38% HCl, and 40 μL EDC (N-(3-dimethylaminopropyl)-N′-ethylcarbodiimide; 5.6 M; Sigma). Reaction was preceded for 3 h or longer (overnight is preferred) at room temperature before the chemicals were washed off from the solid support with 500 μL of 10% formic acid (3×), 500 μL of 10% acetonitrile (3×), 500 μL of 1 M NaCl (3×) and finally H2O (3×).

#### N-Glycan Release

After removing the wash buffer, 2 μL of PNGase F, 16 μL G7 (10×), and 142 μL DI was added to the bead mixture and incubated at 37 °C for overnight to release N-glycans. The eluted glycans were purified by Carbograph and SGP glycans were eluted in 1 mL 80% ACN (0.1% formic acid) while other N-glycan eluents were dried in vacuum^28^. The N-glycans were re-suspended in HPLC grade of water, 200 μL for 1 mg fetuin, 100 μL for serum and 20 μL for CHO respectively.

#### QUANTITIY labeling protocol

The dried glycans were re-suspended in reaction solution mixture consisting of anhydrous dimethyl sulfoxide (DMSO) and acetic acid (AA) (7:3, vol) in the presence of 1 M NaCNBH3. For fetuin, glycans enriched from respective GIG were placed in four vials with a theoretical ratio of 1:1:3:5 (starting fetuin 200:200:600:1000 μg). A fixed volume (20:20:60:100) μL of 100 mM QUANTITY (176, 177, 178, and 179) dissolved in the mixture of DMSO and AA (7:3) were added into each sample respectively and incubated at 65 °C for 4 h. The reaction was quenched by addition of 2 mL water and 2 μL concentrated formic acid. Samples that were labeled with 4-plex QUANTITY were pooled for cleanup by Carbograph. The purified samples were dissolved in 400 μL of 0.2% formic acid.

Glycans released from 1 mg SGP were purified by Carbograph and eluted in 1000 μL of 80% ACN (0.1% TFA). 40 μL eluent was used for labeling with QUANTITY. Another 40 μL SGP was also labeled with aminoxyTMTzero using same protocol as that of QUANTITIY (unfortunately, we did follow manufacturer’s protocol to label glycan with TMT. Results showed only a small amount of glycan conjugated. So far, method using DMSO-HOAc showed best yield). Serum glycans (from 20 μL) were labeled using same protocol as that of SGP.

Glycans were quantitatively characterized by MALDI and ESI-MS. The details on experiments and synthesis of QUANTITY are available in supporting materials.

## Additional Information

**How to cite this article**: Yang, S. *et al.* QUANTITY: An Isobaric Tag for Quantitative Glycomics. *Sci. Rep.* **5**, 17585; doi: 10.1038/srep17585 (2015).

## Supplementary Material

Supplementary Information
