# Supplementary material for: QUANTITY: An Isobaric Tag for Quantitative Glycomics
Source: Sci Rep. 2015 Nov 30;5:17585. doi: 10.1038/srep17585 (PMC4663469; doi:10.1038/srep17585)
Supplement: Supplementary Information [file srep17585-s1.pdf]

QUANTITY: An Isobaric Tag for Quantitative Glycomics

Shuang Yang, Meiyao Wang, Lijun Chen, Bojiao Yin, Guoqiang Song, Illarion V. Turko,

Karen W. Phinney, Michael J. Betenbaugh, Hui Zhang, and Shuwei Li

Contents

Synthesis of QUANTITY 3

Protein tryptic digestion from beads 7

ESI-LC-MS/MS method 8

MALDI-MS/MS analysis method 9

Results 10

1. Glycan quantitation linearity using fetuin and QUANTITY 10
2. Comparison of glycan labeling with QUANTITY and aminoxyTMT-126 11
3. QUANTITY-labelled N-glycans from human sera 12
4. Structure assignment of a precursor ion 13
5. Profiling of serum N-glycans by LC-ESI-MS/MS 16
6. Quantitative profiling of N-glycans from CHO cell lines 20
7. Examples of MS/MS spectra of QUANTITY-labeled N-glycans 27
8. ^{1}H and ^{13}C spectra of two key compounds: compound 9 and 13 30

References 32

Synthesis of QUANTITY

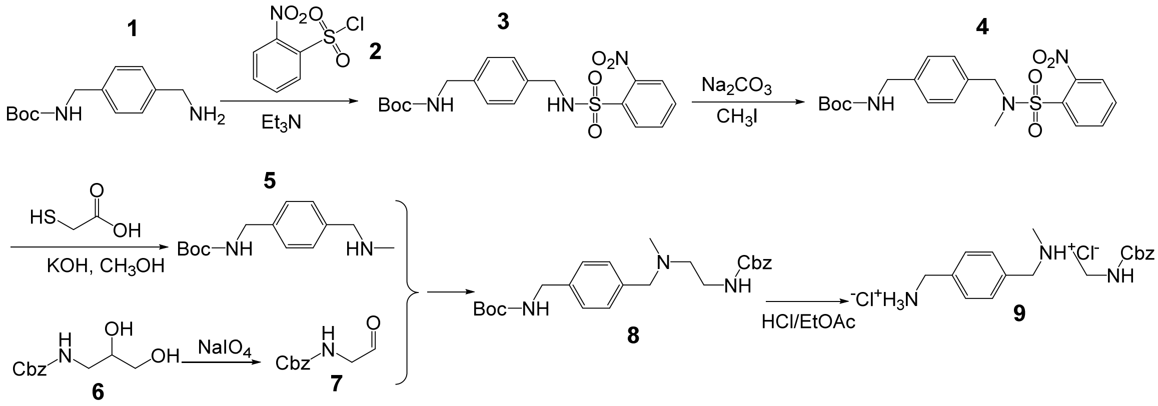

Figure S1. Scheme of chemical synthesis of QUANTITY intermediates. 1 = Boc-(4-(aminomethyl)benzyl)-amine; 2 = 2-nitrobenzenesulfonyl chloride.

Boc-(4-(aminomethyl)benzyl)-amine (**1**, 1.12 g, 4.75 mmol) and 1.5 mL triethylamine (10.8 mmol) were mixed in 30 mL DCM, then 2-nitrobenzenesulfonyl chloride (**2**, 1 g, 4.52 mmol) was added (Figure S1). The reaction was stirred under argon balloon for overnight. After reaction was done, 30 mL more DCM was added. The mixture was washed with 50 mL HCl (50 mM) solution twice, 50 mL saturated NaHCO3 solution twice, and 50 Ml brine solution once. The organic layer was dried with anhydrous Na2SO4 and removed by Rotavap to offer 1.7 g white solid (**3**, 4.03 mmol, 89% yield). **1H-NMR (CDCl3, 400 MHz)** δ 7.60-8.00 (m, 4H), 7.10-7.20 (m, 4H), 4.28 (s, 2H), 4.22 (d, J = 6.0Hz, 2H), 1.45 (s, 9H). **13C-NMR (CDCl3, 101 MHz)** δ 155.88, 147.77, 139.01, 134.79, 133.92, 133.47, 132.75, 131.00, 128.15, 127.61, 125.22, 79.59, 47.54, 44.13, 28.41. **ESI-MS** $(M + H)^+$ = 422.14, Cal. $(M + H)^+$ = 422.14.

Compound **3** (1.7 g, 4.40 mmol) was dissolved in 10 mL DMF and 2.8 g Na2CO3 (26.4 mmol) solid was added. 1.37 mL methyl iodide (22 mmol) was then added. The reaction was stirred in dark for 2 h. The reaction was checked by HPLC for completion. After reaction was done, most CH3I and DMF were removed by Rotavap. The residue was dissolved in 50 mL EtOAc and 50 mL brine solution. The organic layer was

separated and washed by 60 mL brine solution three times, dried over Na2SO4 and removed by Rotavap to offer 1.8 g yellowish solid (**4**, 4.13 mmol, 94% yield). **1H-NMR (CDCl3, 400 MHz)** δ 7.60-8.00 (m, 4H), 7.23-7.30 (m, 4H), 4.39 (s, 2H), 4.30 (d, J = 6.0Hz, 2H), 2.76 (s, 3H), 1.45 (s, 9H). **13C-NMR (CDCl3, 101 MHz)** δ 155.91, 148.25, 138.98, 134.40, 133.61, 132.36, 131.63, 131.02, 128.57, 127.79, 124.17, 79.59, 53.70, 44.28, 34.09, 28.41. **ESI-MS** $(M + H)^+$ = 436.13, Cal. $(M + H)^+$ = 436.14.

Compound **4** (2.56 g, 5.89 mmol) was dissolved in 200 mL 0.5 M KOH/CH3OH solution. 1 mL β-mercaptoacetic acid (14.4 mmol) was added. The reaction was stirred under argon balloon for overnight. After the reaction was done, there was white precipitation in the reaction mixture. After the precipitation was filtered out, methanol was removed carefully by Rotavap. The residue was added with 120 mL water and extracted with 40 mL EtOAc three times. The pooled organic layer was washed by 120 mL saturated NaHCO3 solution once, brine solution 120 mL once. The organic layer was dried over Na2SO4 and removed by Rotavap to offer S3 1.0 g light yellow solid (**5,** 4 mmol, 68% yield). **1H-NMR (CDCl3, 400 MHz)** δ 7.22-7.30 (m, 4H), 4.29 (d, J = 6.0 Hz, 2H), 3.73 (s, 2H), 2.44 (s, 3H), 1.46 (s, 9H). **13C-NMR (CDCl3, 101 MHz)** δ 155.90, 139.02, 137.72, 128.47, 127.57, 79.59, 55.61, 44.43, 35.86, 28.42. **ESI-MS** $(M + H)^+$ = 251.17, Cal. $(M + H)^+$ = 251.18.

Compound **6** (1.8 g, 8 mmol) was dissolved in 100 mL H2O and 2.56 g NaIO4 (12 mmol) was then added. The reaction was stirred for 1 h, extracted with 40 mL DCM three times. The organic layer was washed with 80 mL brine solution once, dried over Na2SO4, and removed by Rotavap. The resulting compound **7** was used without purification for next reaction.

Compound **5** (1 g, 4 mmol) was dissolved in 12 mL MeOH and 400 μL glacial acetic acid was added. 620 mg NaCNBH3 (10 mmol) was dissolved in 4 mL MeOH and added slowly. Then, freshly prepared compound **7** was dissolved in 4 mL MeOH

and added into the mixture slowly. The reaction was stirred at room temperature for 1 h. After reaction was done, most MeOH was removed by Rotavap. The residue was added with 20 mL half saturated $NaHCO_3$ solution, extracted with 20 mL EtOAc three times. The pooled organic layer was washed with 20 mL brine solution once, dried over Na2SO4 and removed by Rotavap to offer 1.54 g oily crude compound **8** (3.6 mmol, 90% yield) **1H-NMR (CDCl3, 400 MHz)** δ 7.30-7.40 (m, 5H), 7.10-7.25 (m, 4H), 5.10 (s, 2H), 4.29 (s, 2H), 3.45 (s, 2H), 3.28 (t, J = 2.0Hz, 2H), 2.48 (t, J = 2.0Hz, 2H), 2.19 (s, 3H), 1.45 (s, 9H). **13C-NMR (CDCl3, 101 MHz)** δ 156.47, 155.94, 137.69, 136.68, 135.80, 129.31, 129.22, 128.61, 128.57, 128.53, 128.20, 128.10, 128.05, 127.48, 79.59, 67.53, 66.64, 55.79, 44.40, 41.70, 38.32, 28.43. **ESI-MS** $(M + H)^+$ = 428.26, Cal. $(M + H)^+$ = 428.25.

Compound **8** (1.54 g) was added with 5 mL DCM and 5 mL trifluoroacetic acid (TFA), incubated for 1 hour. The solvent was removed by Rotavap. 25 mL HCl solution (50 mM) was added. The aqueous layer was washed with 30 mL ether three times. 20 mL saturated K_2CO_3 solution was then added to adjust pH~11. The aqueous layer was extracted with 30 mL EtOAc three times. The organic layer was washed with 30 mL brine solution once, dried over Na_2SO_4 and removed by Rotavap. The resulting oily product was added with 12 mL 1M HCl/EtOAc solution. Precipitation was immediately visible. Solvent was removed by Rotavap to offer light yellowish compound **9** (1.0 g, 70% yield), **which was the key intermediate for the synthesis of all isotope labeled QUANTITY reagents**. **1H-NMR (CD3OD, 400 MHz)** δ 7.55-7.70 (m, 4H), 7.30-7.41 (m, 5H), 5.13 (s, 2H), 4.63 (d, J = 13.2Hz, 1H), 4.35 (d, J = 13.2Hz, 1H), 4.20 (s, 2H), 3.60 (m, 2H), 3.35 (m, 2H), 2.87 (s, 3H). **13CNMR (CD3OD, 101 MHz)** δ 157.8, 135.17, 131.71, 130.22, 129.57, 128.16, 127.80, 127.63, 66.61, 58.89, 55.59, 42.41, 39.42, 35.57. **ESI-MS** $(M + H)^+$ = 328.20, Cal. $(M + H)^+$ = 328.20.

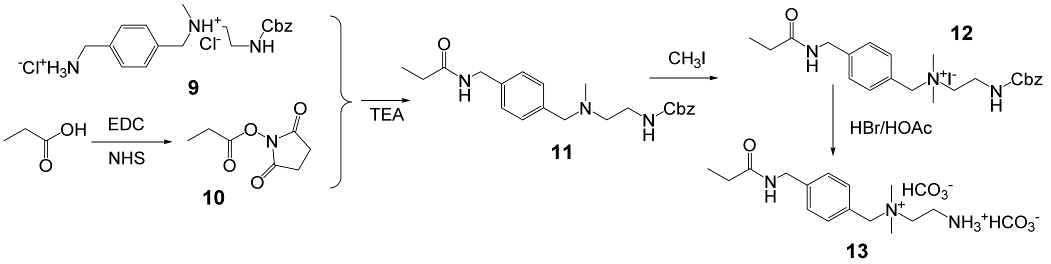

Figure S2. Synthesis of QUANTITIY tag from Intermediate compound **9**.

240 µL propionic acid (3.1 mmol) was dissolved in 20 mL acetonitrile containing 1.65 mL TEA (12.6 mmol). 1.1 g TSTU (3.72 mmol) dissolved in 20 mL acetonitrile was added. The reaction was stirred for 1 hour, then the solvent was removed by Rotavap to offer compound **10,** which was used without purification. Crude compound **10** was mixed with 1.0 g compound **9** (2.5 mmol) in 20 mL anhydrous DMF and 1.65 mL TEA (6.3 mmol) was added. The reaction was stirred for 1 hour until compound **9** was completely consumed. 1 mL 2.5 M K_2CO_3 solution and 15 mL H2O were added to hydrolyze excess compound **10** for 2 h. Most DMF and water were removed by Rotavap. The residue was dissolved in 50 mL EtOAc, washed with 60 mL half saturated $NaHCO_3$ solution three times, 60 mL brine once. The organic layer was dried over Na_2SO_4 and removed by Rotavap to offer compound **11**, which was used without further purification. For non-labeled compound **11**, **1H-NMR (CDCl3, 400 MHz)** δ 7.30-7.40 (m, 5H), 7.17-7.27 (m, 4H), 5.09 (s, 2H), 4.41 (d, J = 6Hz, 2H), 3.47 (s, 2H), 3.28 (m, 2H), 2.49 (m, 2H), 2.23 (q, J = 8.0Hz, 2H), 2.18 (s, 3H), 1.17 (t, J = 8.0Hz, 3H). **13CNMR (CDCl3, 101 MHz)** δ 173.59, 156.42, 138.00, 137.30, 136.69, 129.28, 128.53, 128.10, 127.84, 66.60, 61.94, 55.81, 43.34, 41.77, 38.34, 29.70, 9.88. **ESI-MS** $(M + H)^+$ = 384.23, Cal. $(M + H)^+$ = 384.23.

Crude compound **11** from last step was dissolved in 20 mL anhydrous DMF. 439 µL CH3I (7 mmol) and 1.0 g Na_2CO_3 were added. The reaction was stirred for 2 h. Once the reaction was completed, DMF was removed by Rotavap. The solid was

washed with 20 mL acetonitrile twice. The pooled organic layer was removed by Rotavap to give crude compound **12**, which was added with 20 mL HBr/HOAc and incubated for 1 h. HBr/HOAc was then removed in Ratovap. The residue was dissolved in 20 mL water, washed with 20 mL ether twice, and purified by SCX column (solvent A: 25% acetonitrile, solvent B: 25% acetonitrile, 1 M NH4HCO3). The collected fractions were pooled together and lyophilized. The residual solid was lyophilized in H_2O multiple times to remove NH_4HCO_3 until the weight became constant. The final white solid was 0.57 g (compound **13**, 59% yield). For non-labeled compound **13**, **1H-NMR (CD3OD, 400 MHz)** δ 7.40-7.60 (m, 4H), 4.55 (d, J = 4Hz, 2H), 4.43 (s, 2H), 3.60 (t, J = 6.8Hz, 1H), 3.39 (t, J =6.8Hz, 1H), 3.16 (t, J = 6.8Hz, 2H), 3.07 (d, J = 4.8Hz, 6H), 2.29 (q, J = 7.6Hz, 2H), 1.17 (t, J = 7.6Hz, 3H). **13C-NMR (D_2O, 101 MHz)** δ 178.10, 163.76 (HCO_3 - counterion), 141.05, 133.20, 127.57, 125.85, 68.30, 63.40, 49.79, 42.47, 35.45, 29.14, 9.55. **ESI-MS** $(M + H)^+$ = 264.20, Cal. $(M + H)^+$ = 264.21.

The synthesis of isotope labeled QUANTITY reagents was the same as that of unlabeled compound **13**, but started from the following four pairs of isotope labeled molecules: (CH_3CH_2COOH, $^{13}CHD_2I$); (CH_3CH_2 $^{13}COOH$, CHD_2I); ($^{13}CH_3$ $^{13}CH_2COOH$, $^{13}CH_3I$); ($^{13}CH_3$ $^{13}CH_2$ $^{13}COOH$, CH_3I).

Protein tryptic digestion from beads

The deglycosylated proteins on the solid support were dissolved with 500 µL of 8 M urea in 0.8 M ammonium bicarbonate (NH_4HCO_3; pH 8.0), as described in our recent studies [2]. Proteins on beads were reduced with 10 mM of tris (2-carboxyethyl) phosphine hydrochloride (TCEP) at 37°C for 1 h, followed by alkylation with 20 mM of iodoacetamide (IAA) at room temperature for 30 mins. Samples were then diluted 5-fold with 0.2 M NH_4HCO_3 to have 1.6 M urea before being digested with trypsin at 37°C overnight at a ratio of 1:40 (trypsin:protein). Peptides in 0.1% TFA were purified by C18 3cc Vac Cartridge (500 mg sorbent; Waters Corporation, Milford, MA) and eluted with

500 µL 60% acetonitrile (ACN), 2× (Fisher Scientific, Pittsburgh, PA). Peptides whose concentration was determined by NanoDrop were labeled with four channels of iTRAQ (AB SCIEX, Framingham, MA). The iTRAQ labeled peptides were pooled for C18 cleanup and optionally separated to 8 fractions by basic reverse phase liquid chromatography (bRPLC) on the 1220 Infinity LC system with a Zorbax Extended-C18 analytical column (1.8 µm particles, 4.6 × 100 mm; Agilent Technologies, Inc., CA) [3]. Flow rate is set at 0.2 mL/min and a linear gradient (8 to 35% within 85 min) is used to elute peptide fractions (Buffer A: 10 mM NH_4HCO_2, pH 10; Buffer B: 10 mM NH_4HCO_2 and 90% ACN, pH 10). Each fraction was analyzed on a RPLC mass spectrometer (Details refer to LC-MS analysis) using a Q Exactive Quadrupole for global proteomics (Thermo).

ESI-LC-MS/MS method

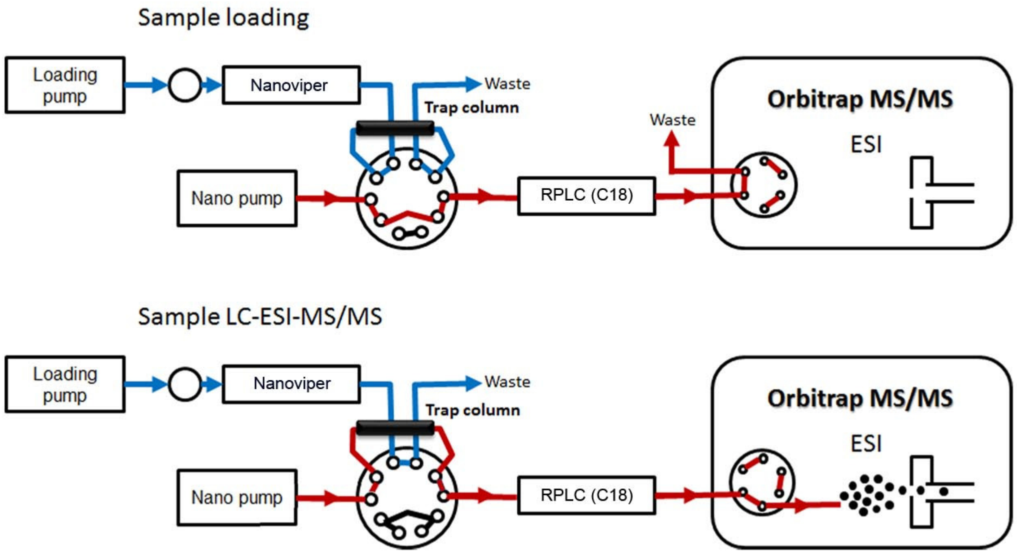

Figure S3. The schematic diagram of LC-ESI-MS/MS on sample loading and separation. The trap column and analytical column are C18,

LC-MS experiments were performed on an Orbitrap LC-ESI-MS/MS. The setup is shown in SI Figure 3. QUANTITY-labeled glycans was dissolved in 13 µl of 0.2% TFA for analysis using Orbitrap (Velos Pro Mass Spectrometer; Thermo Fisher Scientific Inc.; Waltham, MA). The instrumental setup is given in Figure S3. Flow rate was set to 2 µl/min and each test was run for 10 min. The instrument was operated in data-dependent mode with m/z ranging from 300-2000 Da, in which a full MS scan (mass resolution = 60,000) was followed by ten MS/MS scans. The normalized collision energy of higher energy collisional dissociation was 35%, and the dynamic exclusion duration was 25 µs. Ions without assigned charge states were rejected for MS/MS analysis. The heated capillary was maintained at 200°C, while the ESI voltage was maintained at +2.2 kV. Each sample was analyzed in triplicate on the Orbitrap. System control was achieved using Thermo Xcalibur data acquisition software and data analysis was performed with Thermo Xcalibur Qual Browser (2.2 SP1.48). Glycan identification was performed using GlycoWorkbench software (version 2.0) and CFG Functional Glycomics Gateway. The reporter ions were output using program of Matlab.

MALDI-MS/MS analysis method

Glycans (5 µL for each sample out of 1000 µL of elution after Carbograph cleanup) extracted from SGP, Fetuin, serum, and CHO cell lines using GIG were analyzed by Axima MALDI Resonance mass spectrometer (Shimadzu). Matrix solution consists of 4 µL DMA in 200 µL DHB (100 µg/µL in 50% acetonitrile, 0.1 mM NaCl) in that DMA can increase the detection of sialylated glycans. The DHB-DMA (1 µL) spots formed uniform crystals and increased sialylated glycan stability by increasing laser power absorption and ionization efficiency [4]. The laser power was set to be able to detect intact signal (typically 100-140) for 2 shots each in 100 locations per spot. The average MS spectra (200 profiles) were used for glycan assignment by comparing to

the database of glycans previously analyzed by MALDI-MS/MS in our lab. We confirm the assigned glycans from human serum established in literature [5-7].

Results

1. Glycan quantitation linearity using fetuin and QUANTITY

Table S1. Quantitation of N-glycans from bovine fetuin serum using GIG and QUANTITY. SD = standard deviation.

Symbol	F	N	H	S	M	176		177		178		179	
						Average	SD	Average	SD	Average	SD	Average	SD
N_2H_2S	0	2	2	1	2255.0	0.94	0.02	1.06	0.02	3.39	0.11	5.45	0.24
$N_2H_2S_2$	0	2	2	2	2635.2	0.94	0.04	1.06	0.04	3.48	0.13	5.74	0.52
$N_3H_3S_2$	0	3	3	2	3000.4	0.95	0.02	1.05	0.02	3.58	0.21	5.83	0.36
$N_3H_3S_3$	0	3	3	3	3380.5	0.92	0.07	1.08	0.07	3.54	0.36	6.12	0.95
$N_3H_3S_4$	0	3	3	4	3760.7	0.99	0.15	1.01	0.15	3.06	0.35	4.86	0.63

2. *Comparison of glycan labeling with QUANTITY and aminoxyTMT-126*

Figure S4. MALDI-MS spectra of glycan labeled by QUANTITY and aminoxyTMT-126. (a) native SGP N-glycans extracted from GIG; (b) QUANTITY-labelled SGP N-glycans; (c) aminoxyTMT-126 labelled SGP N-glycans.

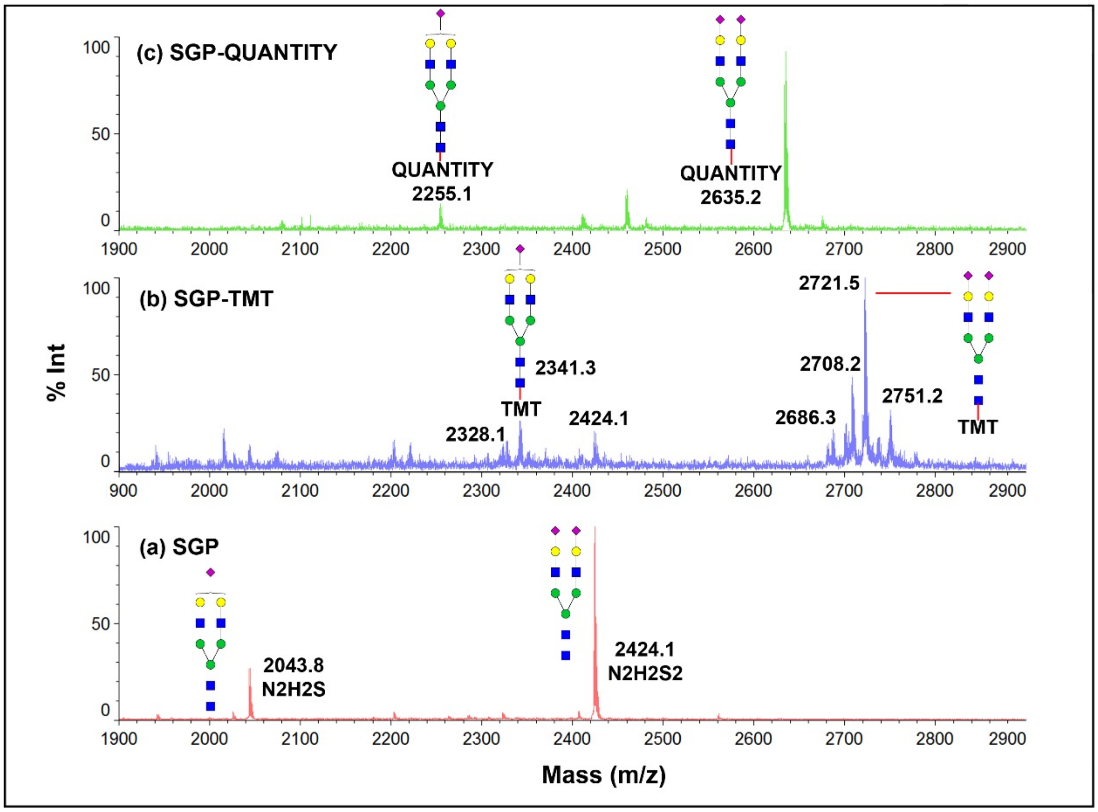

3. QUANTITY-labelled N-glycans from human sera

Figure S5. MALDI-MS spectra of serum native N-glycans (a) and QUANTITY-labeled N-glycans (b) extracted from human sera using GIG.

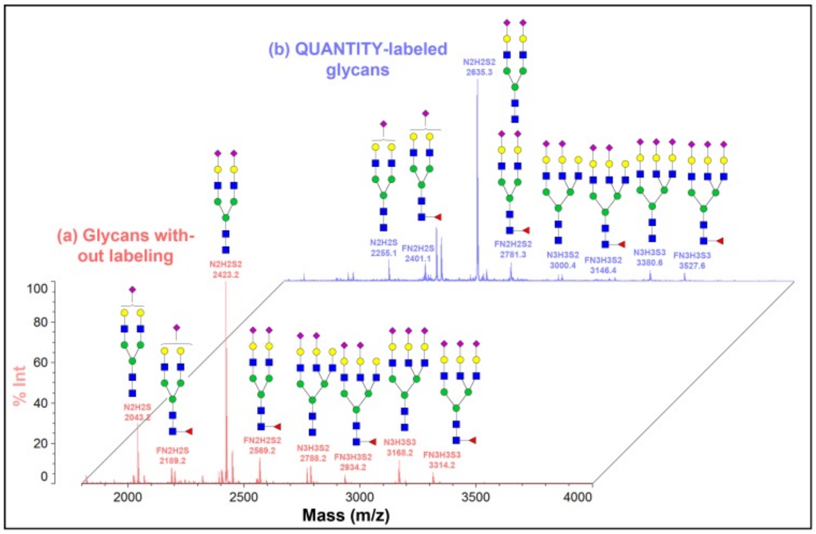

We illustrated complete labeling of serum N-glycan by QUANTITY using MALDI-MS (Figure S5).The profile of glycans in (b) is similar to (a) after QUANTITY labeling.

4. *Structure assignment of a precursor ion*

Figure S6. MS/MS fragments of a precursor ion (1317.57, z = 2) in Orbitrap LC-ESI-MS/MS.

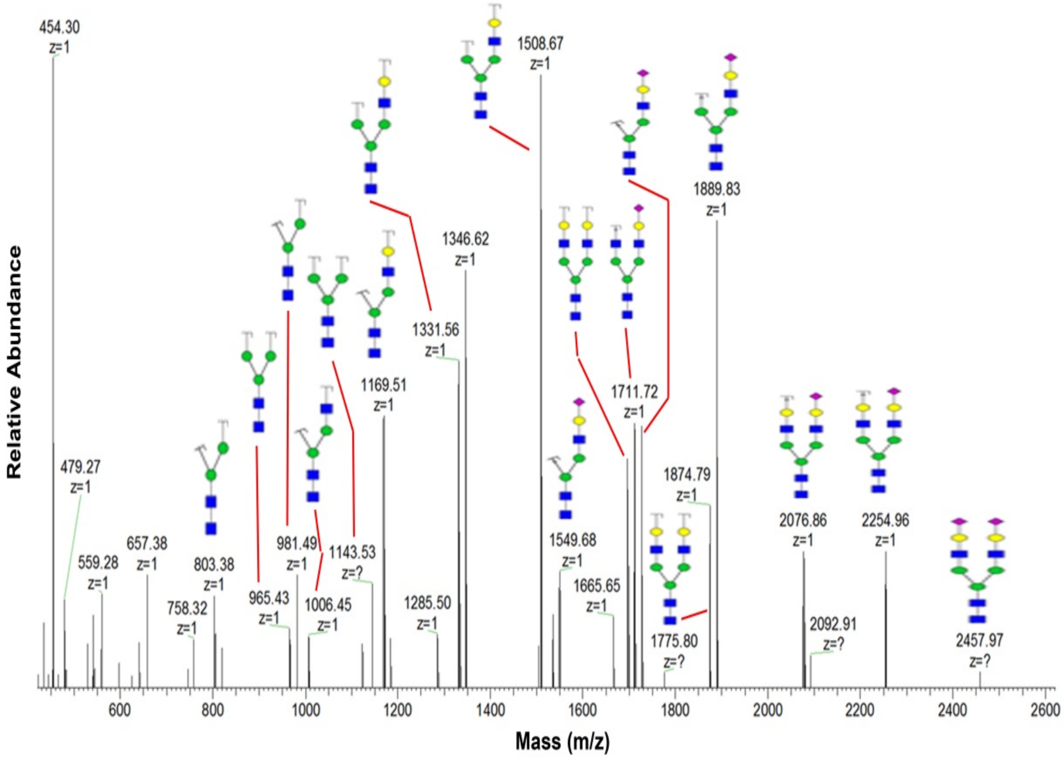

An example of MS/MS spectrum from a precursor ion 1317.57 (z = 2) was shown in Figure S6. The fragments of a precursor ion 1317.57 (z = 2) were shown in SI Table 2.Same methodology has been used for structure assignment to other glycans based on MS/MS spectrum from ESI-MS/MS.

Table S2. Structure assignment of a precursor ion 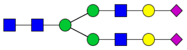 1317.57 (z = 2;) based on MS/MS spectrum. MS/MS spectrum is shown in Figure S6. (Symbol: S = Sialic Acid; Q = QUANTITY reporter ion; N = HexNAc; H = Hexose)

Fragment (m/z and z)	Tag form (1)(2)(3)	Fragment structure	Loss of fragment ions from a precursor ion
803.38 (z = 1)	Tertiary amine	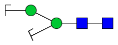	2S, 1Q, 2N, 3H
965.43 (z = 1)	Tertiary amine	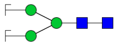	2S, 1Q, 2N, 2H
981.49 (z = 1)	Intact	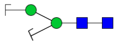	2S, 2N, 3H
1006.45 (z = 1)	Tertiary amine	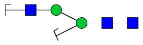	2S, 1Q, 1N, 3H
1143.53	Intact	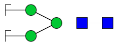	2S, 2N, 2H
1169.51 (z = 1)	Tertiary amine	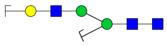	2S, 1Q, 1N, 2H
1331.56 (z = 1)	Tertiary amine	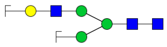	2S, 1Q, 1N, 1H
1508.67 (z = 1)	Intact	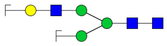	2S, 1N, 1H
1549.68 (z = 1)	Tertiary amine	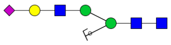	1S, 1Q, 1N, 2H
1696.69 (z = 1)	Tertiary amine	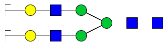	2S, 1Q
1711.72 (z = 1)	Tertiary amine	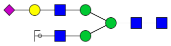	1S, 1Q, 1N, 1H
1727.78 (z = 1)	Intact	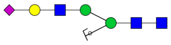	1S, 1N, 2H
1874.79 (z = 1)	Intact	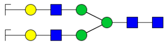	2S
1889.83 (z = 1)	Intact	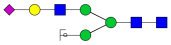	1S, 1N, 1H
2076.86 (z = 1)	Tertiary amine	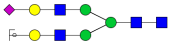	1S, 1Q
2254.96 (z = 1)	Intact	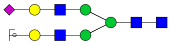	1S
2457.97	Tertiary amine	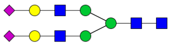	1Q

Note: (1) Only QUANTITY tag coupled fragments are visible in MS2 spectra. Upon MS/MS fragmentation, a QUANTITY tag can remain intact or break into one of two forms (carbocation or tertiary amine) as shown below. (2) It is normal if a fragment containing the tertiary amine form of the QUANTITY tag has a mass shift of 1-3 Da away from the calculated monoisotopic mass of this fragment. This mass shift is due to the balancer that is left in the tertiary amine form (Figure S7). (3) The generation of tertiary amine and carbocation ions seems to be exclusive as we can observe either tertiary amine or carbocation series fragments, but not both, on the same MS2 spectrum.

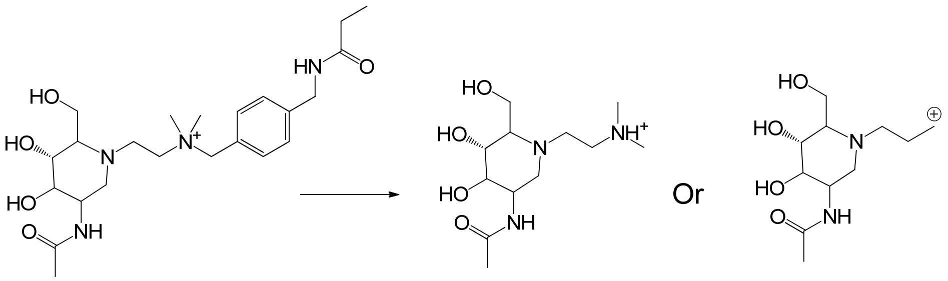

Intact form Tertiary amine form Carbocation form

QUANTITY or its fragments attached to a GlcNAc

Figure S7. Intact QUANTITY or its fragments attached to a GlcNAc.

Because of significantly increased hydrophobicity of QUANTITY-labeled glycans, the ionization has been improved. We tested the identification of glycans from human serum. Over 90 glycans have been identified as shown in Table S2; the MS spectrum of human serum was shown in Figure S7.

5. *Profiling of serum N-glycans by LC-ESI-MS/MS*

Table S3. Profiling of QUANTITIY-labeled N-glycans from human sera by ESI-MS/MS (Note: core structure is not shown in the Table).

Symbol	F	N	H	S	G	G-pT	$[M + H]^{+}$	$[M + H]^{2+}$	$[M + H]^{3+}$	$[M + H]^{4+}$
	a	b	c	d						
N	0	1	0	0	1136.4	1136.4	1347.7	673.8	449.2	336.9
FH	1	0	1	0	1241.4	1241.4	1452.7	726.3	484.2	363.2
FH_2	1	0	2	0	1403.5	1403.5	1614.7	807.4	538.2	403.7
NS	0	1	0	1	1449.5	1516.6	1727.8	863.9	575.9	432.0
N_3	0	3	0	0	1542.6	1542.6	1753.8	876.9	584.6	438.5
NHS	0	1	1	1	1611.6	1678.7	1889.9	944.9	630.0	472.5
FNHS	1	1	1	1	1757.6	1824.7	2035.9	1018.0	678.6	509.0
N_2HS	0	2	1	1	1814.7	1881.8	2093.0	1046.5	697.7	523.2
N_2H_2S	0	2	2	1	1976.7	2043.8	2255.0	1127.5	751.7	563.8
N_3HS	0	3	1	1	2017.8	2084.8	2296.1	1148.0	765.4	574.0
$F_3N_2H_2$	3	2	2	0	2101.8	2101.8	2313.0	1156.5	771.0	578.3
H_5S	0	0	5	1	2056.7	2123.8	2335.0	1167.5	778.3	583.8
$F_2N_3H_2$	2	3	2	0	2158.8	2158.8	2370.1	1185.0	790.0	592.5
FN_2H_2S	1	2	2	1	2122.8	2189.9	2401.1	1200.5	800.4	600.3
NH_2S_2	0	1	2	2	2086.7	2220.9	2432.1	1216.1	810.7	608.0
N_3H_2S	0	3	2	1	2179.8	2246.9	2458.1	1229.1	819.4	614.5
N_4HS	0	4	1	1	2220.9	2287.9	2499.2	1249.6	833.1	624.8
F_4N_3H	4	3	1	0	2288.9	2288.9	2500.1	1250.1	833.4	625.0
FN_3H_2S	1	3	2	1	2325.9	2393.0	2604.2	1302.1	868.1	651.0
N_3H_3S	0	3	3	1	2341.9	2409.0	2620.2	1310.1	873.4	655.0
$N_2H_2S_2$	0	2	2	2	2289.8	2424.0	2635.2	1317.6	878.4	658.8
FN_6H	1	6	1	0	2460.0	2460.0	2671.2	1335.6	890.4	667.8

$F_3N_3H_3$	3	3	3	0	2466.9	2466.9	2678.2	1339.1	892.7	669.5
$F_3N_2H_2S$	3	2	2	1	2414.9	2482.0	2693.2	1346.6	897.7	673.3
$F_2N_3H_4$	2	3	4	0	2482.9	2482.9	2694.2	1347.1	898.1	673.5
$F_2N_3H_2S$	2	3	2	1	2471.9	2539.0	2750.2	1375.1	916.7	687.6
$FN_2H_2S_2$	1	2	2	2	2435.9	2570.0	2781.3	1390.6	927.1	695.3
$N_2H_3S_2$	0	2	3	2	2451.9	2586.0	2797.3	1398.6	932.4	699.3
$N_3H_2S_2$	0	3	2	2	2492.9	2627.1	2838.3	1419.2	946.1	709.6
NH_7S	0	1	7	1	2583.9	2651.0	2862.2	1431.1	954.1	715.6
$F_3N_3H_2S$	3	3	2	1	2618.0	2685.1	2896.3	1448.2	965.4	724.1
$FN_3H_2S_2$	1	3	2	2	2639.0	2773.1	2984.4	1492.2	994.8	746.1
$N_3H_3S_2$	0	3	3	2	2655.0	2789.1	3000.4	1500.2	1000.1	750.1
$FN_3H_3S_2$	1	3	3	2	2801.0	2935.2	3146.4	1573.2	1048.8	786.6
$FN_4H_2S_2$	1	4	2	2	2842.1	2976.2	3187.5	1593.7	1062.5	796.9
F_2N_9	2	9	0	0	3053.3	3053.3	3264.5	1632.3	1088.2	816.1
$F_2N_3H_3S_2$	2	3	3	2	2947.1	3081.2	3292.5	1646.2	1097.5	823.1
$F_2N_2H_2S_3$	2	2	2	3	2895.0	3096.3	3307.5	1653.8	1102.5	826.9
$N_4H_4S_2$	0	4	4	2	3020.1	3154.3	3365.5	1682.8	1121.8	841.4
$N_3H_3S_3$	0	3	3	3	2968.1	3169.3	3380.5	1690.3	1126.8	845.1
$F_3N_2H_4S_2$	3	2	4	2	3052.1	3186.3	3397.5	1698.7	1132.5	849.4
$F_3N_4H_4S$	3	4	4	1	3145.2	3212.3	3423.5	1711.8	1141.2	855.9
$F_3N_3H_3S_2$	3	3	3	2	3093.1	3227.3	3438.5	1719.3	1146.2	859.6
$F_3N_2H_2S_3$	3	2	2	3	3041.1	3242.3	3453.6	1726.8	1151.2	863.4
$F_2N_4H_3S_2$	2	4	3	2	3150.2	3284.3	3495.6	1747.8	1165.2	873.9
$FN_4H_4S_2$	1	4	4	2	3166.2	3300.3	3511.6	1755.8	1170.5	877.9
$FN_3H_3S_3$	1	3	3	3	3114.1	3315.4	3526.6	1763.3	1175.5	881.7
$N_3H_4S_3$	0	3	4	3	3130.1	3331.4	3542.6	1771.3	1180.9	885.6
$N_5H_4S_2$	0	5	4	2	3223.2	3357.4	3568.6	1784.3	1189.5	892.2
$N_4H_3S_3$	0	4	3	3	3171.2	3372.4	3583.6	1791.8	1194.5	895.9

FN_9H_3	1	9	3	0	3393.4	3393.4	3604.6	1802.3	1201.5	901.2
$F_2N_5H_5S$	2	5	5	1	3364.3	3431.4	3642.6	1821.3	1214.2	910.6
$F_2N_4H_4S_2$	2	4	4	2	3312.2	3446.4	3657.6	1828.8	1219.2	914.4
$F_2N_3H_3S_3$	2	3	3	3	3260.2	3461.4	3672.7	1836.3	1224.2	918.2
$FN_4H_3S_3$	1	4	3	3	3317.2	3518.5	3729.7	1864.8	1243.2	932.4
$N_4H_4S_3$	0	4	4	3	3333.2	3534.5	3745.7	1872.8	1248.6	936.4
$N_3H_3S_4$	0	3	3	4	3281.2	3549.5	3760.7	1880.4	1253.6	940.2
$N_5H_3S_3$	0	5	3	3	3374.2	3575.5	3786.7	1893.4	1262.2	946.7
$F_3N_4H_4S_2$	3	4	4	2	3458.3	3592.5	3803.7	1901.8	1267.9	950.9
$FN_5H_5S_2$	1	5	5	2	3531.3	3665.5	3876.7	1938.4	1292.2	969.2
$FN_4H_4S_3$	1	4	4	3	3479.3	3680.5	3891.7	1945.9	1297.2	972.9
$FN_3H_3S_4$	1	3	3	4	3427.2	3695.5	3906.8	1953.4	1302.3	976.7
$FN_5H_3S_3$	1	5	3	3	3520.3	3721.6	3932.8	1966.4	1310.9	983.2
$F_2N_6H_6S$	2	6	6	1	3729.4	3796.5	4007.7	2003.9	1335.9	1001.9
$F_2N_4H_4S_3$	2	4	4	3	3625.3	3826.6	4037.8	2018.9	1345.9	1009.5
$N_5H_5S_3$	0	5	5	3	3698.4	3899.6	4110.8	2055.4	1370.3	1027.7
$N_4H_4S_4$	0	4	4	4	3646.3	3914.6	4125.9	2062.9	1375.3	1031.5
$F_3N_3H_5S_3$	3	3	5	3	3730.3	3931.6	4142.8	2071.4	1380.9	1035.7
$F_4N_4H_3S_3$	4	4	3	3	3755.4	3956.6	4167.9	2083.9	1389.3	1042.0
$F_3N_4H_4S_3$	3	4	4	3	3771.4	3972.6	4183.9	2091.9	1394.6	1046.0
$F_4N_5H_2S_3$	4	5	2	3	3796.4	3997.7	4208.9	2104.5	1403.0	1052.2
$F_3N_5H_3S_3$	3	5	3	3	3812.4	4013.7	4224.9	2112.5	1408.3	1056.2
$FN_6H_6S_2$	1	6	6	2	3896.5	4030.6	4241.9	2120.9	1414.0	1060.5
$FN_5H_5S_3$	1	5	5	3	3844.4	4045.7	4256.9	2128.4	1419.0	1064.2
$FN_4H_4S_4$	1	4	4	4	3792.4	4060.7	4271.9	2136.0	1424.0	1068.0
$N_4H_5S_4$	0	4	5	4	3808.4	4076.7	4287.9	2144.0	1429.3	1072.0
FN_9H_5S	1	9	5	1	4030.6	4097.7	4308.9	2154.5	1436.3	1077.2
$F_2N_4H_4S_4$	2	4	4	4	3938.4	4206.8	4418.0	2209.0	1472.7	1104.5

$N_6H_6S_3$	0	6	6	3	4063.5	4264.8	4476.0	2238.0	1492.0	1119.0
$N_5H_5S_4$	0	5	5	4	4011.5	4279.8	4491.0	2245.5	1497.0	1122.8
$F_3N_4H_4S_4$	3	4	4	4	4084.5	4352.8	4564.0	2282.0	1521.3	1141.0
$FN_5H_5S_4$	1	5	5	4	4157.5	4425.8	4637.1	2318.5	1545.7	1159.3
$F_2N_5H_5S_4$	2	5	5	4	4303.6	4571.9	4783.1	2391.6	1594.4	1195.8
$N_6H_6S_4$	0	6	6	4	4376.6	4644.9	4856.2	2428.1	1618.7	1214.0
$F_3N_5H_5S_4$	3	5	5	4	4449.6	4718.0	4929.2	2464.6	1643.1	1232.3
$F_2N_6H_6S_4$	2	6	6	4	4668.7	4937.0	5148.3	2574.1	1716.1	1287.1
$N_7H_7S_4$	0	7	7	4	4741.7	5010.1	5221.3	2610.7	1740.4	1305.3
$N_6H_6S_5$	0	6	6	5	4689.7	5025.1	5236.3	2618.2	1745.4	1309.1
$F_3N_9H_3S_4$	3	9	3	4	4937.9	5206.2	5417.5	2708.7	1805.8	1354.4
$N_6H_6S_6$	0	6	6	6	5002.8	5405.3	5616.5	2808.3	1872.2	1404.1

Figure S8. Profiling of QUQNAITY-labeled N-glycan extracted from CHO cell lines using GIG by Orbitrap LC-ESI-MS/MS.

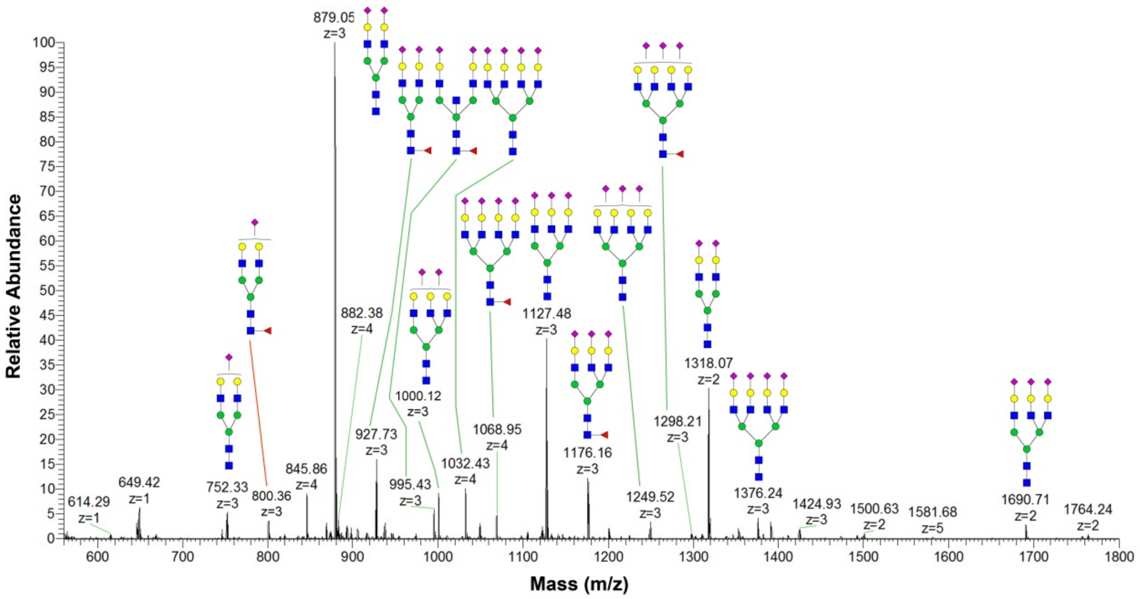

6. *Quantitative profiling of N-glycans from CHO cell lines*

Table S4. List of QUANTITY-labeled glycans from the pooled CHO cell lines. CHO WT is labeled with QUANTITY 176, CHO.ST6 with 178, and CHO.ST3Gal4 with 179.

List	Symbol	F	N	H	S	G	G-pT	M	WT	ST6Gal1	ST3Gal4
		a	b	c	d					(+)	(-)
1	H_2	0	0	2	0	1257.4	1257.4	1468.7	0.00E+00	0.00E+00	0.00E+00
2	FN	1	1	0	0	1282.5	1282.5	1493.7	1.86E-03	4.74E-03	4.48E-03
3	NH	0	1	1	0	1298.5	1298.5	1509.7	1.36E-03	1.90E-02	3.39E-03
4	F_2H	2	0	1	0	1387.5	1387.5	1598.7	0.00E+00	0.00E+00	0.00E+00
5	H_3	0	0	3	0	1419.5	1419.5	1630.7	0.00E+00	0.00E+00	0.00E+00
6	FNH	1	1	1	0	1444.5	1444.5	1655.8	2.97E-03	3.84E-03	3.25E-03
7	N_2H	0	2	1	0	1501.6	1501.6	1712.8	0.00E+00	1.32E-02	0.00E+00
8	NS	0	1	0	1	1449.5	1516.6	1727.8	1.24E-02	0.00E+00	8.38E-03
9	F_3H	3	0	1	0	1533.6	1533.6	1744.8	0.00E+00	3.97E-02	0.00E+00
10	FH_3	1	0	3	0	1565.5	1565.5	1776.8	1.80E-03	0.00E+00	0.00E+00
11	H_4	0	0	4	0	1581.5	1581.5	1792.8	0.00E+00	7.62E-03	0.00E+00
12	FNH_2	1	1	2	0	1606.6	1606.6	1817.8	0.00E+00	1.03E-02	0.00E+00
13	FHS	1	0	1	1	1554.5	1621.6	1832.9	0.00E+00	9.99E-03	0.00E+00
14	FN_2H	1	2	1	0	1647.6	1647.6	1858.9	0.00E+00	1.90E-02	0.00E+00
15	FNS	1	1	0	1	1595.6	1662.7	1873.9	6.40E-02	1.08E-02	4.22E-02
16	N_2H_2	0	2	2	0	1663.6	1663.6	1874.9	1.12E-02	1.34E-02	1.07E-02
17	NHS	0	1	1	1	1611.6	1678.7	1889.9	9.90E-03	4.54E-02	5.31E-03
18	FN_3	1	3	0	0	1688.7	1688.7	1899.9	0.00E+00	0.00E+00	0.00E+00
19	N_3H	0	3	1	0	1704.7	1704.7	1915.9	0.00E+00	5.40E-03	1.54E-02
20	FN_2H_2	1	2	2	0	1809.7	1809.7	2020.9	1.40E-02	4.73E-03	2.15E-02
21	FNHS	1	1	1	1	1757.6	1824.7	2035.9	9.48E-03	1.54E-02	7.28E-03
22	NH_2S	0	1	2	1	1773.6	1840.7	2051.9	1.68E-03	2.41E-02	1.83E-03

23	N_2HS	0	2	1	1	1814.7	1881.8	2093.0	6.63E-03	1.90E-02	3.56E-03
24	FNH_2S	1	1	2	1	1919.7	1986.8	2198.0	9.23E-03	1.70E-02	1.01E-02
25	NH_3S	0	1	3	1	1935.7	2002.8	2214.0	0.00E+00	0.00E+00	0.00E+00
26	FN_2HS	1	2	1	1	1960.7	2027.8	2239.0	9.06E-03	9.61E-03	7.02E-03
27	N_3H_3	0	3	3	0	2028.8	2028.8	2240.0	1.39E-03	1.00E-02	1.30E-03
28	N_2H_2S	0	2	2	1	1976.7	2043.8	2255.0	9.15E-03	6.30E-02	9.39E-03
29	FN_4H	1	4	1	0	2053.8	2053.8	2265.1	0.00E+00	0.00E+00	0.00E+00
30	NHS_2	0	1	1	2	1924.7	2058.8	2270.1	0.00E+00	4.40E-02	0.00E+00
31	F_3NH_3	3	1	3	0	2060.8	2060.8	2272.0	0.00E+00	1.82E-02	0.00E+00
32	H_7	0	0	7	0	2067.7	2067.7	2278.9	1.98E-03	0.00E+00	3.68E-03
33	N_3HS	0	3	1	1	2017.8	2084.8	2296.1	0.00E+00	1.02E-02	0.00E+00
34	FN_5	1	5	0	0	2094.9	2094.9	2306.1	1.11E-02	1.19E-02	8.47E-03
35	N_5H	0	5	1	0	2110.9	2110.9	2322.1	8.93E-04	5.48E-02	4.57E-03
36	F_3NHS	3	1	1	1	2049.7	2116.8	2328.1	0.00E+00	0.00E+00	0.00E+00
37	F_2N_2HS	2	2	1	1	2106.8	2173.9	2385.1	0.00E+00	4.34E-03	0.00E+00
38	FN_2H_2S	1	2	2	1	2122.8	2189.9	2401.1	2.86E-02	5.53E-02	3.01E-02
39	$FNHS_2$	1	1	1	2	2070.7	2204.9	2416.1	0.00E+00	3.21E-02	1.67E-03
40	N_2H_3S	0	2	3	1	2138.8	2205.9	2417.1	0.00E+00	2.64E-02	0.00E+00
41	FN_3HS	1	3	1	1	2163.8	2230.9	2442.1	2.06E-03	0.00E+00	0.00E+00
42	F_2N_5	2	5	0	0	2240.9	2240.9	2452.2	1.80E-02	1.49E-02	2.30E-02
43	FN_5H	1	5	1	0	2256.9	2256.9	2468.1	0.00E+00	4.33E-02	0.00E+00
44	FN_3H_4	1	3	4	0	2336.9	2336.9	2548.1	0.00E+00	2.47E-02	0.00E+00
45	FN_3H_2S	1	3	2	1	2325.9	2393.0	2604.2	0.00E+00	4.52E-03	0.00E+00
46	N_3H_3S	0	3	3	1	2341.9	2409.0	2620.2	1.96E-03	9.62E-03	1.05E-03
47	$N_2H_2S_2$	0	2	2	2	2289.8	2424.0	2635.2	1.80E-02	5.37E-02	1.35E-02
48	F_2N_6	2	6	0	0	2444.0	2444.0	2655.2	0.00E+00	1.66E-02	0.00E+00
49	FN_6H	1	6	1	0	2460.0	2460.0	2671.2	0.00E+00	6.19E-03	0.00E+00
50	N_3HS_2	0	3	1	2	2330.9	2465.0	2676.3	1.63E-02	0.00E+00	1.48E-02

51	N_6H_2	0	6	2	0	2476.0	2476.0	2687.2	2.11E-02	6.01E-02	1.80E-02
52	$F_2N_3H_4$	2	3	4	0	2482.9	2482.9	2694.2	0.00E+00	0.00E+00	0.00E+00
53	F_4N_4H	4	4	1	0	2492.0	2492.0	2703.2	1.36E-03	2.66E-02	4.26E-03
54	$F_2N_2H_3S$	2	2	3	1	2430.9	2498.0	2709.2	0.00E+00	3.44E-03	0.00E+00
55	FN_7	1	7	0	0	2501.1	2501.1	2712.3	0.00E+00	4.80E-03	0.00E+00
56	$F_3N_4H_2$	3	4	2	0	2508.0	2508.0	2719.2	0.00E+00	2.03E-02	0.00E+00
57	FN_4H_4	1	4	4	0	2540.0	2540.0	2751.2	0.00E+00	2.71E-02	0.00E+00
58	FN_3H_3S	1	3	3	1	2487.9	2555.0	2766.2	0.00E+00	9.83E-03	0.00E+00
59	N_4H_5	0	4	5	0	2556.0	2556.0	2767.2	2.37E-03	7.13E-03	3.63E-03
60	$FN_2H_2S_2$	1	2	2	2	2435.9	2570.0	2781.3	4.51E-02	1.15E-01	3.65E-02
61	N_3H_4S	0	3	4	1	2503.9	2571.0	2782.2	1.77E-03	1.62E-02	1.81E-03
62	$F_4N_2H_4$	4	2	4	0	2572.0	2572.0	2783.2	2.35E-03	9.12E-03	1.61E-03
63	$N_2H_3S_2$	0	2	3	2	2451.9	2586.0	2797.3	8.77E-03	3.60E-02	9.26E-03
64	$F_3N_2H_5$	3	2	5	0	2588.0	2588.0	2799.2	0.00E+00	0.00E+00	0.00E+00
65	FN_4H_2S	1	4	2	1	2529.0	2596.1	2807.3	1.01E-02	7.42E-02	1.55E-02
66	N_4H_3S	0	4	3	1	2545.0	2612.0	2823.3	1.53E-03	1.17E-02	5.56E-03
67	FN_6H_2	1	6	2	0	2622.1	2622.1	2833.3	4.80E-02	5.04E-03	4.27E-02
68	$N_3H_2S_2$	0	3	2	2	2492.9	2627.1	2838.3	1.66E-03	1.45E-02	6.11E-03
69	$N_3H_3S_2$	0	3	3	2	2655.0	2789.1	3000.4	1.07E-03	0.00E+00	5.84E-03
70	FN_6HS	1	6	1	1	2773.1	2840.2	3051.4	9.83E-03	2.79E-02	1.31E-02
71	$F_3N_5H_3$	3	5	3	0	2873.1	2873.1	3084.4	6.32E-04	3.29E-02	5.95E-03
72	$F_2N_2H_3S_2$	2	2	3	2	2744.0	2878.2	3089.4	0.00E+00	0.00E+00	0.00E+00
73	$FN_3H_3S_2$	1	3	3	2	2801.0	2935.2	3146.4	2.84E-03	5.71E-03	4.87E-03
74	N_4H_5S	0	4	5	1	2869.1	2936.2	3147.4	6.35E-04	5.95E-03	1.63E-03
75	$FN_2H_2S_3$	1	2	2	3	2749.0	2950.2	3161.5	9.15E-04	6.79E-03	0.00E+00
76	$FN_4H_2S_2$	1	4	2	2	2842.1	2976.2	3187.5	1.15E-03	1.28E-03	8.06E-03
77	$F_4N_4H_4$	4	4	4	0	2978.2	2978.2	3189.4	0.00E+00	0.00E+00	0.00E+00
78	$N_4H_3S_2$	0	4	3	2	2858.1	2992.2	3203.5	7.83E-03	1.54E-02	1.47E-02

79	$F_2N_6H_2S$	2	6	2	1	3081.2	3148.3	3359.5	0.00E+00	5.81E-03	0.00E+00
80	$N_3H_3S_3$	0	3	3	3	2968.1	3169.3	3380.5	5.55E-03	5.92E-03	6.76E-03
81	$N_4H_2S_3$	0	4	2	3	3009.1	3210.4	3421.6	8.92E-03	8.17E-03	1.10E-02
82	$F_3N_4H_4S$	3	4	4	1	3145.2	3212.3	3423.5	0.00E+00	0.00E+00	0.00E+00
83	N_7H_3S	0	7	3	1	3154.2	3221.3	3432.6	6.38E-03	1.71E-02	9.78E-03
84	$F_4N_6H_3$	4	6	3	0	3222.3	3222.3	3433.5	8.97E-03	1.12E-02	1.04E-02
85	$F_3N_2H_2S_3$	3	2	2	3	3041.1	3242.3	3453.6	8.65E-03	6.45E-03	8.55E-03
86	F_4N_6HS	4	6	1	1	3211.3	3278.4	3489.6	5.96E-03	1.18E-02	8.96E-03
87	$F_3N_7H_3$	3	7	3	0	3279.3	3279.3	3490.6	3.75E-03	1.01E-02	6.60E-03
88	$FN_3H_3S_3$	1	3	3	3	3114.1	3315.4	3526.6	1.58E-02	9.00E-03	1.69E-02
89	$FN_5H_3S_2$	1	5	3	2	3207.2	3341.4	3552.6	9.34E-03	4.43E-03	1.22E-02
90	$F_4N_5H_5$	4	5	5	0	3343.3	3343.3	3554.5	7.18E-03	1.47E-02	5.28E-03
91	$FN_4H_2S_3$	1	4	2	3	3155.2	3356.4	3567.6	6.63E-03	1.10E-02	1.25E-02
92	$N_5H_4S_2$	0	5	4	2	3223.2	3357.4	3568.6	7.25E-03	6.68E-03	1.33E-02
93	$F_4N_4H_4S$	4	4	4	1	3291.2	3358.3	3569.6	0.00E+00	0.00E+00	0.00E+00
94	FN_7H_3S	1	7	3	1	3300.3	3367.4	3578.6	0.00E+00	0.00E+00	0.00E+00
95	N_8H_5	0	8	5	0	3368.4	3368.4	3579.6	2.21E-01	1.36E-01	2.64E-01
96	$N_4H_3S_3$	0	4	3	3	3171.2	3372.4	3583.6	0.00E+00	1.86E-02	1.43E-03
97	$N_3H_2S_4$	0	3	2	4	3119.1	3387.4	3598.7	2.64E-03	1.57E-02	6.39E-03
98	$F_4N_2H_2S_3$	4	2	2	3	3187.1	3388.4	3599.6	4.72E-04	1.63E-02	1.97E-03
99	$F_4N_4H_2S_2$	4	4	2	2	3280.2	3414.4	3625.6	4.74E-03	0.00E+00	5.62E-03
100	FN_7HS_2	1	7	1	2	3289.3	3423.5	3634.7	5.56E-03	2.15E-02	9.92E-03
101	N_7H_3S	0	8	3	1	3357.3	3424.4	3635.7	2.21E-03	2.36E-02	4.20E-03
102	$F_4N_7H_3$	4	7	3	0	3425.4	3425.4	3636.6	0.00E+00	0.00E+00	0.00E+00
103	$F_3N_4H_4S_2$	3	4	4	2	3458.3	3592.5	3803.7	3.76E-03	2.47E-02	9.29E-03
104	$F_4N_6H_3S$	4	6	3	1	3535.4	3602.5	3813.7	3.48E-02	2.10E-02	3.38E-02
105	$F_3N_3H_3S_3$	3	3	3	3	3406.2	3607.5	3818.7	8.96E-03	5.04E-03	1.37E-02
106	$F_3N_3H_8S$	3	3	8	1	3590.3	3657.4	3868.6	8.49E-03	7.47E-03	1.11E-02

107	$FN_4H_4S_3$	1	4	4	3	3479.3	3680.5	3891.7	0.00E+00	0.00E+00	0.00E+00
108	$FN_5H_3S_3$	1	5	3	3	3520.3	3721.6	3932.8	2.43E-03	6.43E-03	6.26E-03
109	$F_4N_5H_5S$	4	5	5	1	3656.4	3723.5	3934.7	2.84E-03	1.13E-02	1.19E-02
110	N_9H_6	0	9	6	0	3733.5	3733.5	3944.7	1.72E-02	5.09E-02	3.51E-02
111	$FN_4H_2S_4$	1	4	2	4	3468.3	3736.6	3947.8	1.47E-03	1.07E-02	7.18E-03
112	$N_5H_4S_3$	0	5	4	3	3536.3	3737.6	3948.8	2.14E-03	1.01E-02	3.83E-03
113	$F_4N_4H_4S_2$	4	4	4	2	3604.3	3738.5	3949.7	0.00E+00	2.49E-02	7.50E-03
114	$N_4H_3S_4$	0	4	3	4	3484.2	3752.6	3963.8	3.78E-03	9.60E-03	6.36E-03
115	$F_4N_3H_3S_3$	4	3	3	3	3552.3	3753.5	3964.8	0.00E+00	0.00E+00	5.23E-03
116	$FN_8H_2S_2$	1	8	2	2	3654.4	3788.6	3999.8	2.64E-03	1.17E-02	3.59E-03
117	N_9H_4S	0	9	4	1	3722.5	3789.6	4000.8	0.00E+00	1.15E-02	4.08E-03
118	$F_4N_4H_3S_3$	4	4	3	3	3755.4	3956.6	4167.9	2.25E-03	3.90E-02	8.52E-03
119	$F_3N_5H_5S_2$	3	5	5	2	3823.4	3957.6	4168.8	0.00E+00	2.65E-02	0.00E+00
120	$F_3N_4H_4S_3$	3	4	4	3	3771.4	3972.6	4183.9	4.81E-03	2.34E-02	1.12E-02
121	$FN_5H_5S_3$	1	5	5	3	3844.4	4045.7	4256.9	0.00E+00	0.00E+00	0.00E+00
122	$FN_4H_4S_4$	1	4	4	4	3792.4	4060.7	4271.9	0.00E+00	0.00E+00	0.00E+00
123	$F_2N_6H_3S_3$	2	6	3	3	3869.5	4070.7	4281.9	0.00E+00	1.08E-02	0.00E+00
124	$FN_6H_4S_3$	1	6	4	3	3885.5	4086.7	4297.9	4.04E-03	3.29E-02	1.08E-02
125	$FN_5H_3S_4$	1	5	3	4	3833.4	4101.7	4313.0	1.27E-03	3.17E-02	3.46E-03
126	$N_6H_5S_3$	0	6	5	3	3901.5	4102.7	4313.9	0.00E+00	0.00E+00	0.00E+00
127	$F_4N_5H_5S_2$	4	5	5	2	3969.5	4103.7	4314.9	0.00E+00	1.27E-02	0.00E+00
128	$F_4N_8H_6$	4	8	6	0	4114.6	4114.6	4325.9	2.10E-02	9.72E-02	4.17E-02
129	$N_5H_4S_4$	0	5	4	4	3849.4	4117.7	4329.0	0.00E+00	2.45E-02	7.01E-03
130	$F_4N_4H_4S_3$	4	4	4	3	3917.4	4118.7	4329.9	0.00E+00	9.81E-03	0.00E+00
131	$F_3N_8H_7$	3	8	7	0	4130.6	4130.6	4341.9	5.92E-03	2.26E-02	1.18E-02
132	$F_4N_3H_3S_4$	4	3	3	4	3865.4	4133.7	4345.0	7.22E-04	3.80E-02	5.41E-03
133	$FN_9H_3S_2$	1	9	3	2	4019.6	4153.8	4365.0	4.76E-03	2.79E-02	9.86E-03
134	$N_9H_4S_2$	0	9	4	2	4035.6	4169.8	4381.0	2.07E-03	2.02E-02	6.98E-03

135	$F_4N_8H_4S$	4	8	4	1	4103.6	4170.7	4381.9	4.47E-04	2.63E-02	6.09E-03
136	$F_3N_6H_3S_3$	3	6	3	3	4015.5	4216.8	4428.0	5.91E-04	1.32E-02	3.28E-03
137	$N_6H_4S_4$	0	6	4	4	4052.5	4320.8	4532.1	0.00E+00	0.00E+00	0.00E+00
138	$F_4N_4H_3S_4$	4	4	3	4	4068.5	4336.8	4548.0	0.00E+00	0.00E+00	0.00E+00
139	$F_3N_5H_5S_3$	3	5	5	3	4136.5	4337.8	4549.0	0.00E+00	1.79E-02	5.47E-03
140	$FN_6H_4S_4$	1	6	4	4	4198.6	4466.9	4678.1	2.81E-03	1.86E-02	4.00E-03
141	$F_4N_6H_6S_2$	4	6	6	2	4334.6	4468.8	4680.0	0.00E+00	5.72E-03	0.00E+00
142	$F_4N_9H_7$	4	9	7	0	4479.8	4479.8	4691.0	4.00E-03	4.23E-02	7.83E-03
143	$N_6H_5S_4$	0	6	5	4	4214.5	4482.9	4694.1	2.12E-03	5.26E-02	3.45E-03
144	$F_4N_5H_5S_3$	4	5	5	3	4282.6	4483.8	4695.1	0.00E+00	6.64E-03	0.00E+00
145	$F_4N_4H_4S_4$	4	4	4	4	4230.5	4498.9	4710.1	0.00E+00	1.30E-02	2.34E-03
146	$F_2N_9H_2S_3$	2	9	2	3	4316.7	4517.9	4729.2	2.30E-03	1.37E-02	7.88E-03
147	$FN_9H_3S_3$	1	9	3	3	4332.7	4533.9	4745.2	5.51E-04	1.24E-02	1.20E-03
148	$F_3N_6H_5S_3$	3	6	5	3	4339.6	4540.9	4752.1	0.00E+00	9.48E-03	2.81E-03
149	$F_4N_6H_4S_4$	4	5	4	4	4433.6	4702.0	4913.2	0.00E+00	1.07E-02	0.00E+00
150	$FN_6H_6S_4$	1	6	6	4	4522.7	4791.0	5002.2	0.00E+00	0.00E+00	0.00E+00
151	$FN_7H_5S_4$	1	7	5	4	4563.7	4832.0	5043.3	0.00E+00	9.41E-03	0.00E+00
152	$F_4N_7H_7S_2$	4	7	7	2	4699.8	4834.0	5045.2	1.92E-03	5.11E-03	5.81E-03
153	$F_5N_5H_4S_4$	5	5	4	4	4579.7	4848.0	5059.3	0.00E+00	4.15E-02	3.35E-03
154	$F_4N_5H_5S_4$	4	5	5	4	4595.7	4864.0	5075.2	0.00E+00	8.97E-03	0.00E+00
155	$N_5H_5S_6$	0	5	5	6	4637.6	5040.1	5251.4	0.00E+00	0.00E+00	0.00E+00
156	$F_3N_8H_8S_2$	3	8	8	2	4918.9	5053.0	5264.3	0.00E+00	0.00E+00	0.00E+00
157	$FN_5H_5S_6$	1	5	5	6	4783.7	5186.2	5397.4	0.00E+00	2.72E-02	0.00E+00
158	$FN_7H_5S_5$	1	7	5	5	4876.8	5212.2	5423.4	0.00E+00	2.35E-02	0.00E+00
159	$F_4N_7H_7S_3$	4	7	7	3	5012.9	5214.1	5425.4	0.00E+00	1.13E-02	0.00E+00

Figure S9. Heatmap of relative abundance of non-sialic acid in CHO cell lines (WT, ST6Gal1(+), and ST3Gal4(-))

Symbol	F	N	H	S	G-pT-Q
FN	1	1	0	0	1493.71
NH	0	1	1	0	1509.71
FNH	1	1	1	0	1655.77
N2H	0	2	1	0	1712.80
F3H	3	0	1	0	1744.79
FH3	1	0	3	0	1776.78
H4	0	0	4	0	1792.77
FNH2	1	1	2	0	1817.82
FN2H	1	2	1	0	1858.86
N2H2	0	2	2	0	1874.86
N3H	0	3	1	0	1915.90
FN2H2	1	2	2	0	2020.91
N3H3	0	3	3	0	2240.01
F3NH3	3	1	3	0	2271.99
H7	0	0	7	0	2278.93
FN5	1	5	0	0	2306.10
N5H	0	5	1	0	2322.09
F2N5	2	5	0	0	2452.15
FN5H	1	5	1	0	2468.15
FN3H4	1	3	4	0	2548.12
F2N6	2	6	0	0	2655.25
FN6H	1	6	1	0	2671.24
N6H2	0	6	2	0	2687.24
F4N4H	4	4	1	0	2703.23
FN7	1	7	0	0	2712.29
F3N4H2	3	4	2	0	2719.22
FN4H4	1	4	4	0	2751.21
N4H5	0	4	5	0	2767.21
F4N2H4	4	2	4	0	2783.19
FN6H2	1	6	2	0	2833.30
F3N5H3	3	5	3	0	3084.37
F4N6H3	4	6	3	0	3433.52
F3N7H3	3	7	3	0	3490.56
F4N5H5	4	5	5	0	3554.53
N8H5	0	8	5	0	3579.59
N9H6	0	9	6	0	3944.74
F4N8H6	4	8	6	0	4325.87
F3N8H7	3	8	7	0	4341.87
F4N9H7	4	9	7	0	4691.02

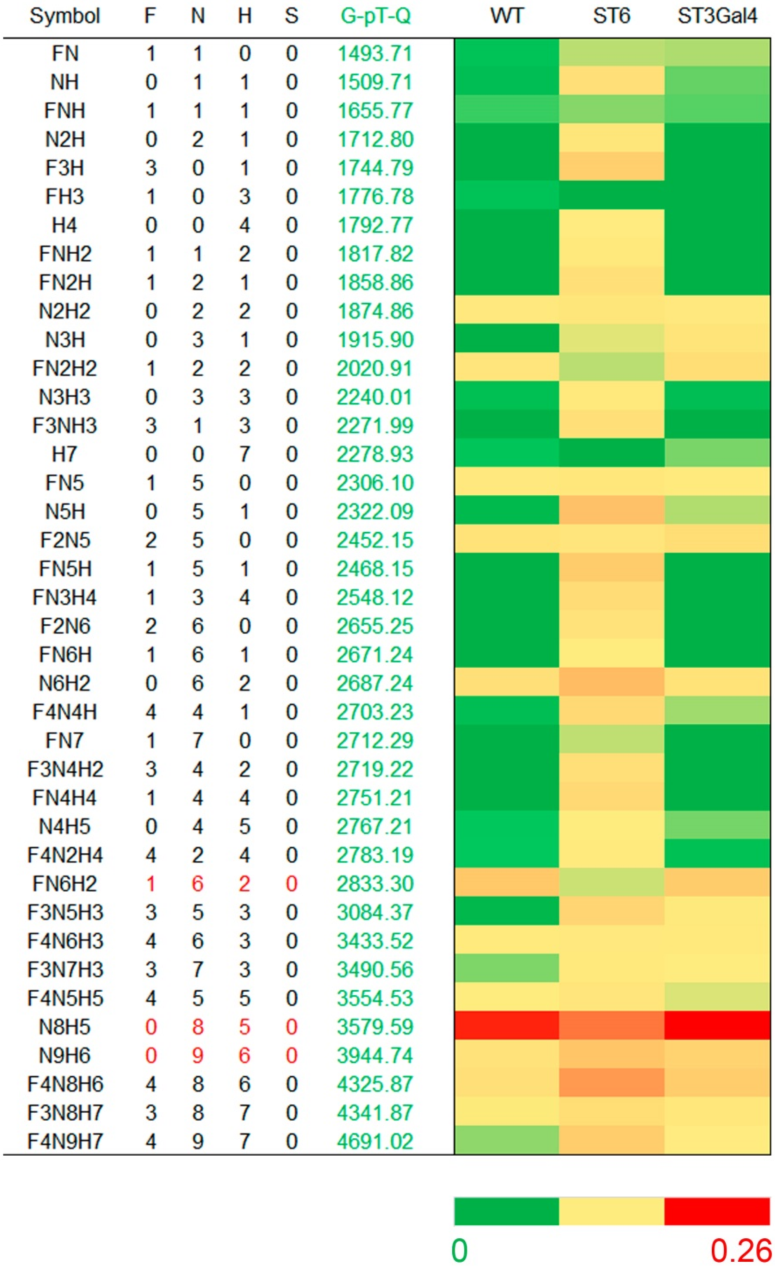

7. *Examples of MS/MS spectra of QUANTITY-labeled N-glycans*

More representative MS/MS spectra of labeled N-glycans (1)

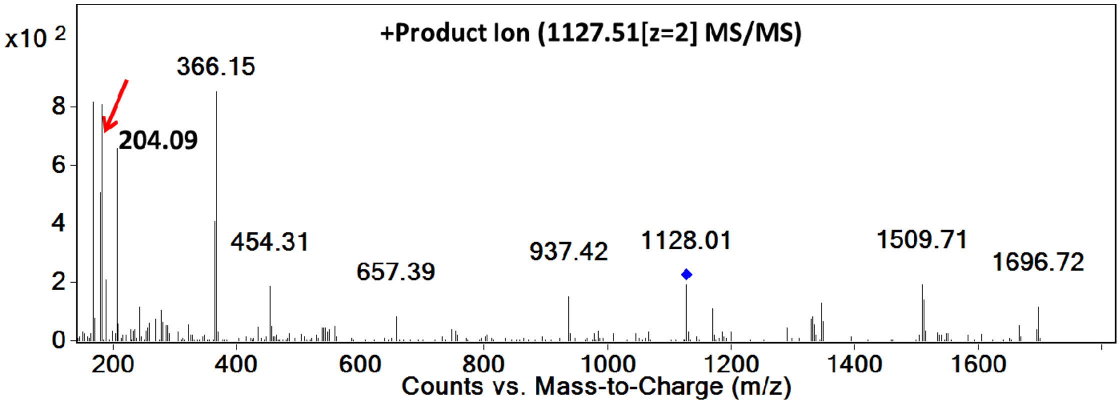

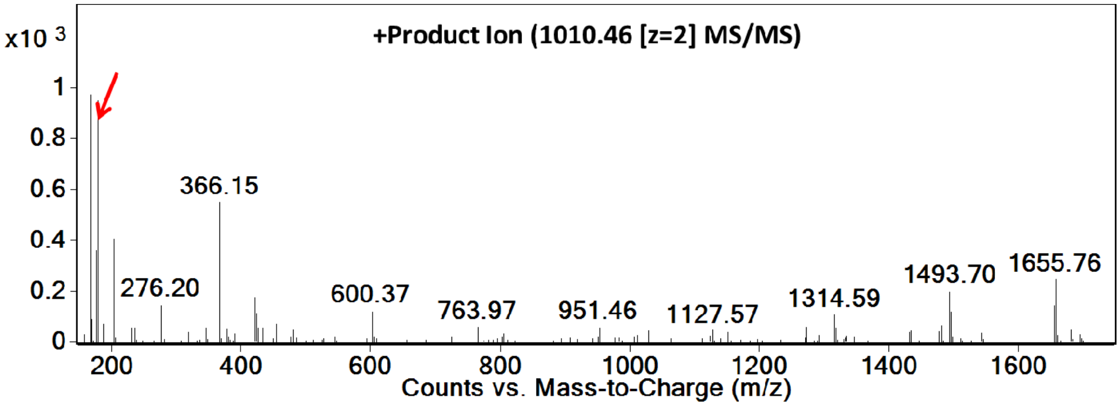

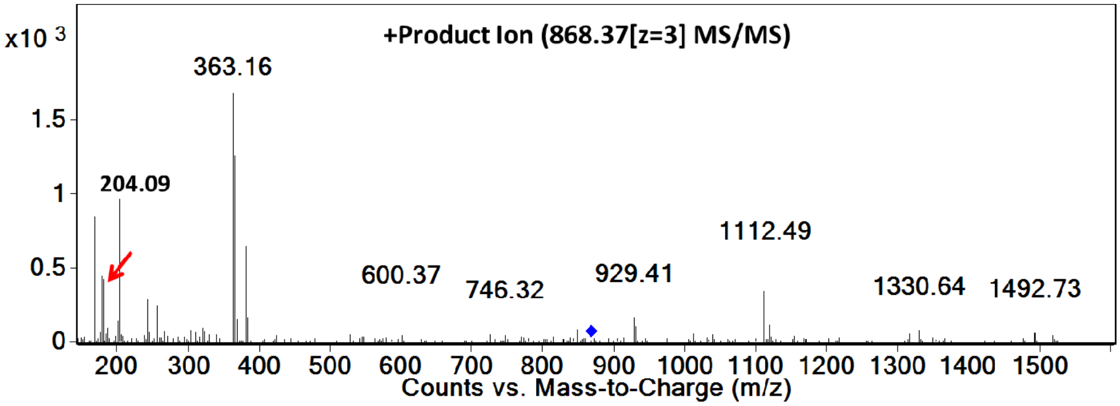

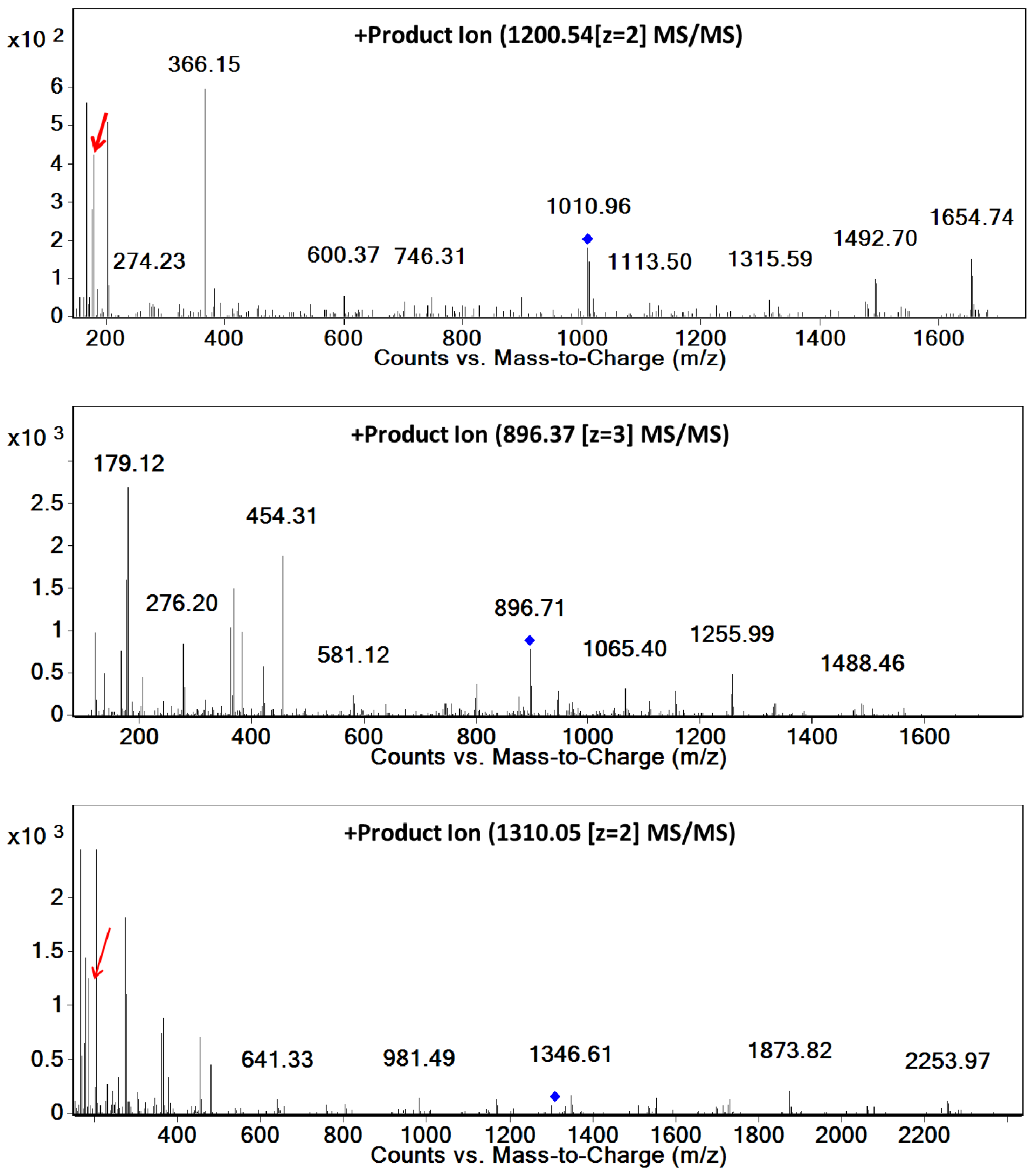
+Product Ion (1200.54[z=2] MS/MS)
x10 2
366.15
274.23
600.37
746.31
1010.96
1113.50
1315.59
1492.70
1654.74
Counts vs. Mass-to-Charge (m/z)
+Product Ion (896.37 [z=3] MS/MS)
x10 3
179.12
454.31
276.20
581.12
896.71
1065.40
1255.99
1488.46
Counts vs. Mass-to-Charge (m/z)
+Product Ion (1310.05 [z=2] MS/MS)
x10 3
641.33
981.49
1346.61
1873.82
2253.97
Counts vs. Mass-to-Charge (m/z)

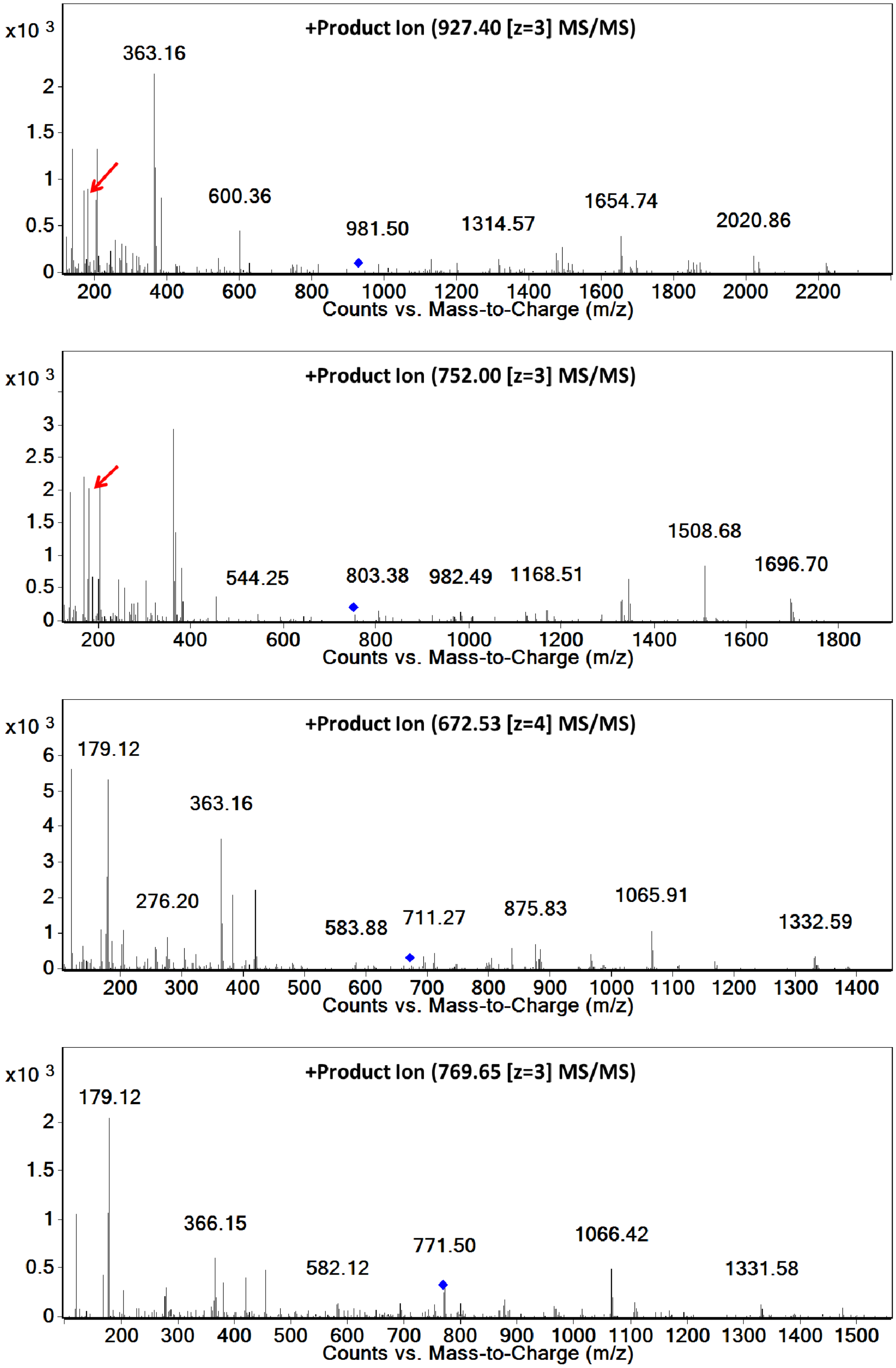

Note: (1) reporter ions (176, 177, 178, 179) are indicated with a red arrow.

8. ***^{1}H and ^{13}C spectra of two key compounds: compound 9 and 13***

Compound 9

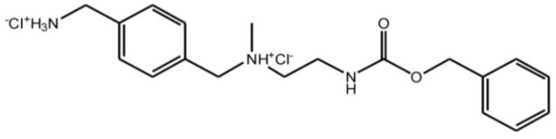

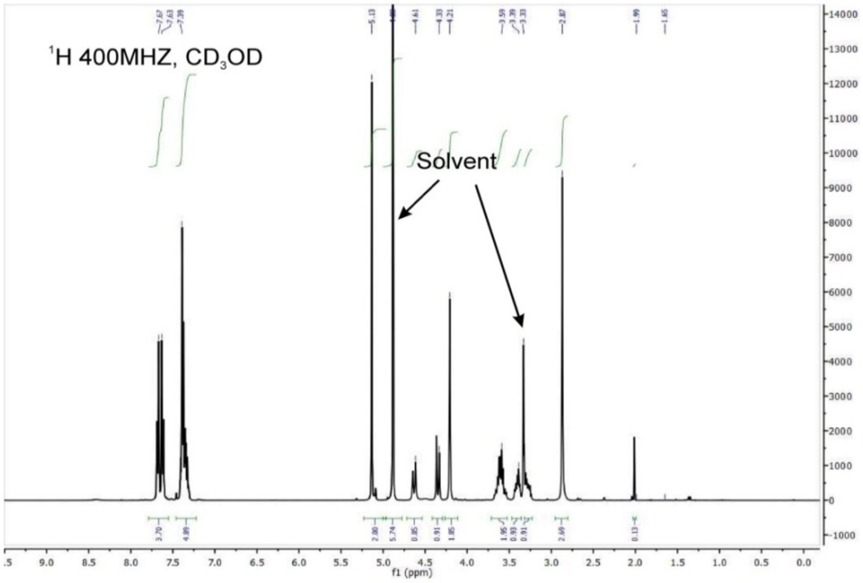

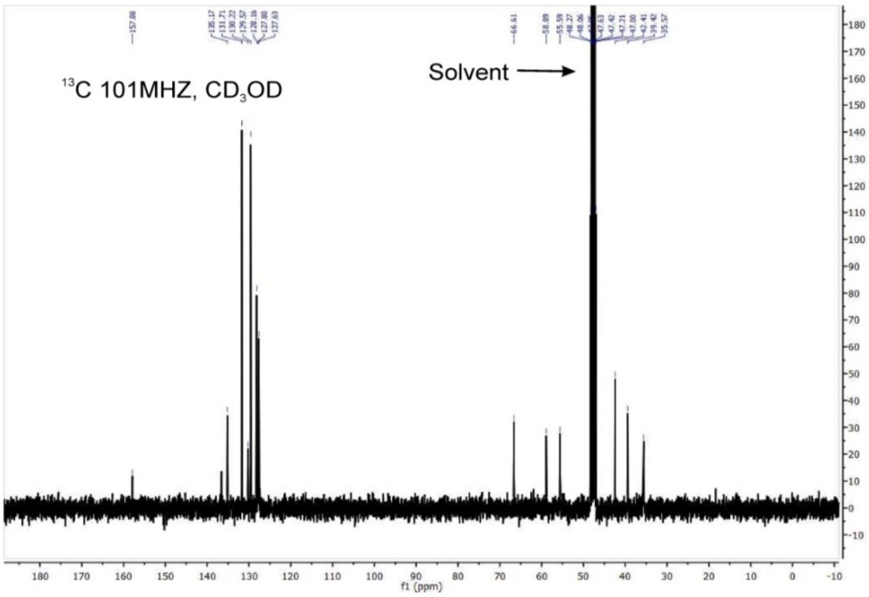

Compound 13

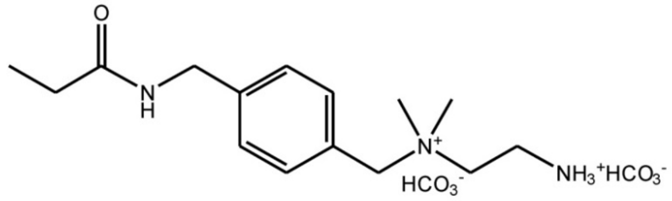
O
N
H
N+
HCO3-
NH3+HCO3-

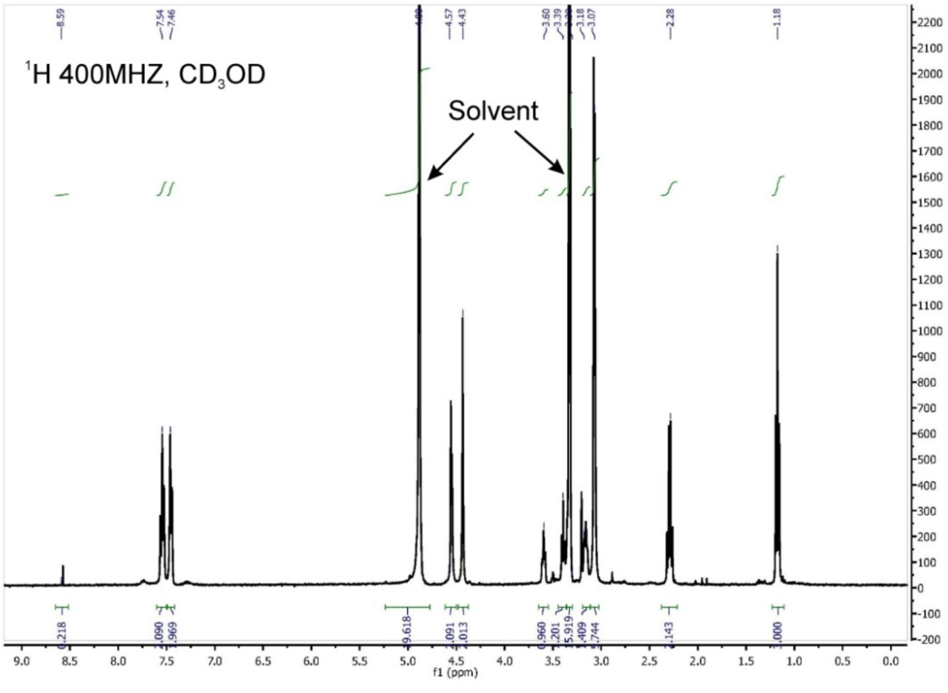
1H 400MHZ, CD3OD
Solvent
8.59
7.54
7.46
4.57
4.43
3.60
3.39
3.18
3.07
2.28
1.18
f1 (ppm)

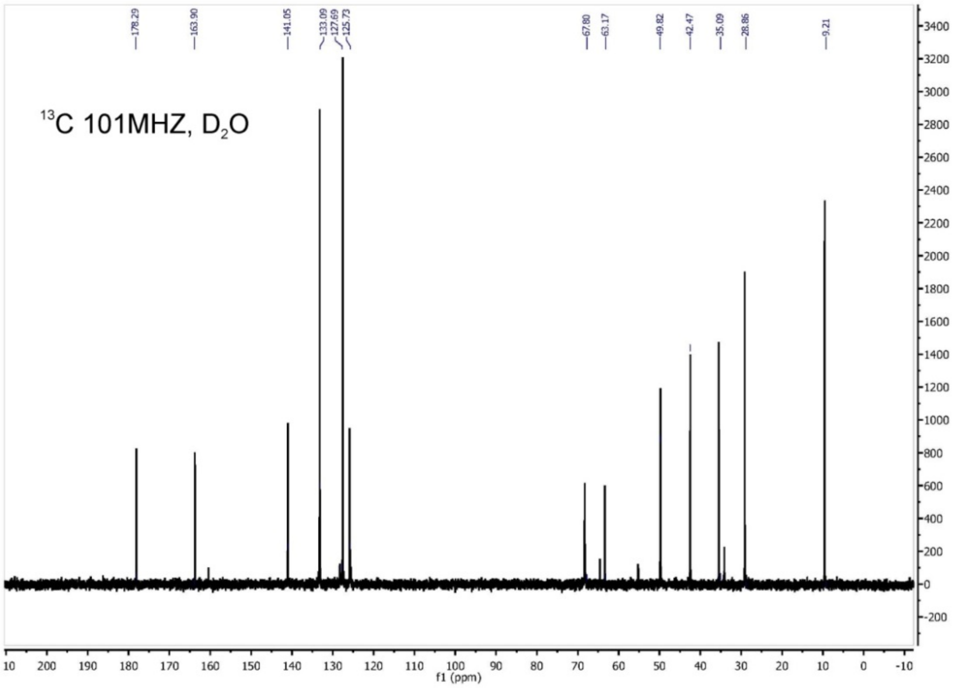
13C 101MHZ, D2O
178.29
163.90
141.05
133.09
127.69
125.73
67.80
63.17
49.82
42.47
35.09
28.86
9.21
f1 (ppm)

References

1. Yang, S. & Zhang, H. Glycan analysis by reversible reaction to hydrazide beads and mass spectrometry. *Anal. Chem.* **84**, 2232-2238 (2012).
2. Yang, S. et al. An Integrated Glycoprotein Immobilization Method for Glycopeptide and Glycan Analysis of Cardiac Hypertrophy. *Anal. Chem.* **Submitted** (2015).
3. Yang, S. et al. Glycoproteins identified from heart failure and treatment models. *Proteomics* **15**, 567-579 (2015).
4. Snovida, S.I. & Perreault, H. A 2, 5-dihydroxybenzoic acid/N, N-dimethylaniline matrix for the analysis of oligosaccharides by matrix-assisted laser desorption/ionization mass spectrometry. *Rapid Commun. Mass Spectrom.* **21**, 3711-3715 (2007).
5. Aldredge, D., An, H.J., Tang, N., Waddell, K. & Lebrilla, C.B. Annotation of a serum N-glycan library for rapid identification of structures. *J. Proteome Res.* **11**, 1958-1968 (2012).
6. Stumpo, K.A. & Reinhold, V.N. The N-glycome of human plasma. *J. Proteome Res.* **9**, 4823-4830 (2010).
7. Miura, Y. et al. BlotGlycoABC™, an integrated glycoblotting technique for rapid and large scale clinical glycomics. *Mol. Cell. Proteomics* **7**, 370-377 (2008).
